# Comparative genome-centric analysis reveals seasonal variation in the function of coral reef microbiomes

**DOI:** 10.1038/s41396-020-0622-6

**Published:** 2020-03-02

**Authors:** Bettina Glasl, Steven Robbins, Pedro R. Frade, Emma Marangon, Patrick W. Laffy, David G. Bourne, Nicole S. Webster

**Affiliations:** 1 Australian Institute of Marine Science, Townsville, QLD Australia; 2 College of Science and Engineering, James Cook University, Townsville, QLD Australia; 3 AIMS@JCU, Townsville, QLD Australia; 4 Australian Centre for Ecogenomics, University of Queensland, Brisbane, QLD Australia; 5 Centre of Marine Sciences, University of Algarve, Faro, Portugal

**Keywords:** Microbial ecology, Water microbiology, Coral reefs

## Abstract

Microbially mediated processes contribute to coral reef resilience yet, despite extensive characterisation of microbial community variation following environmental perturbation, the effect on microbiome function is poorly understood. We undertook metagenomic sequencing of sponge, macroalgae and seawater microbiomes from a macroalgae-dominated inshore coral reef to define their functional potential and evaluate seasonal shifts in microbially mediated processes. In total, 125 high-quality metagenome-assembled genomes were reconstructed, spanning 15 bacterial and 3 archaeal phyla. Multivariate analysis of the genomes relative abundance revealed changes in the functional potential of reef microbiomes in relation to seasonal environmental fluctuations (e.g. macroalgae biomass, temperature). For example, a shift from Alphaproteobacteria to Bacteroidota-dominated seawater microbiomes occurred during summer, resulting in an increased genomic potential to degrade macroalgal-derived polysaccharides. An 85% reduction of Chloroflexota was observed in the sponge microbiome during summer, with potential consequences for nutrition, waste product removal, and detoxification in the sponge holobiont. A shift in the Firmicutes:Bacteroidota ratio was detected on macroalgae over summer with potential implications for polysaccharide degradation in macroalgal microbiomes. These results highlight that seasonal shifts in the dominant microbial taxa alter the functional repertoire of host-associated and seawater microbiomes, and highlight how environmental perturbation can affect microbially mediated processes in coral reef ecosystems.

## Introduction

Coral reef ecosystems are being challenged by anthropogenic pressures that are resulting in unprecedented rates of decline [1–3]. The cumulative effects of climate change (e.g. ocean warming and ocean acidification) and local pressures (e.g. overfishing and eutrophication) reduce the resilience of coral reef ecosystems [4, 5] and lead to a transition from healthy, coral-dominated ecosystems to degraded reefs, often characterised by enhanced macroalgae biomass [6, 7]. The increase of macroalgae in coral reef ecosystems at the expense of coral species abundance and diversity fosters a perpetuating cycle of reef degradation, hence, high macroalgae biomass is often considered a sign of poor reef health [7, 8].

Microorganisms play pivotal roles in coral reefs, and the maintenance of biogeochemical cycling and microbially mediated ecological processes is considered critical for the persistence of reefs under future projected climate conditions [9–11]. Cumulative environmental stressors (e.g. increased sea-surface temperatures, ocean acidification, and eutrophication) can trigger alterations in the composition and function of microbial assemblages associated with corals and sponges [12–16]. Changes in the microbiome of dominant reef-benthos can negatively impact holobiont health, with adverse consequences for the wider reef ecosystem [15, 17, 18]. For example, elevated sea-surface temperatures can disrupt the microbiome of both corals and sponges, leading to disease and mortality [17, 19]. Sponges can comprise a dominant component of the reef benthos, where they form part of a highly efficient recycling pathway that takes up dissolved organic matter and converts it into cellular detritus that becomes food for higher trophic levels [20]. Hence, the break-down of sponge-microbe symbioses can have potential consequences on an ecosystem scale (reviewed by [14]). Furthermore, the transition from coral to macroalgae dominance in reef ecosystems enhances the availability of labile dissolved organic carbon (DOC) in reef waters, shifting the trophic structure towards higher microbial biomass and energy use in degraded reefs, a process termed microbialisation [8, 21]. Macroalgae-derived DOC fosters the growth of copiotrophic, potentially pathogenic, bacterioplankton communities that can negatively impact the health of corals [8, 22–24]. Close proximity of macroalgae to corals can also induce shifts in the coral-associated microbial communities and potentially act as a trigger for microbial diseases [19, 25–27]. As corals perish, more space becomes available for macroalgae, thereby creating a positive feedback loop called DDAM; DOC, disease, algae, microorganism [8, 28].

Metagenomics is providing new insights into the functional roles microorganisms play on coral reefs (e.g. [8, 29, 30]). However, the enormous habitat complexity of coral reefs means that microbial communities associated with different reef niches are rarely holistically assessed within a single study [31]. Given the strong benthic-pelagic coupling that occurs in coral reef ecosystems, integrated functional assessments of free-living and host-associated microbiomes are needed to better understand the contributions of microbially mediated processes to reef ecosystem health [31, 32]. Furthermore, recent computational advances enable precise metabolic reconstructions of microbial genomes from complex microbial communities [33–35]. Thus, identifying how the functional potential of reef microbiomes respond to environmental changes (e.g. temperature and nutrient availability) and benthic species composition (i.e. macroalgae and coral abundance) is now possible at an ecosystem scale.

This genome-centric coral reef microbiome study assessed microbial community shifts in response to seasonal fluctuations in the environment (i.e. sea-surface temperature, macroalgae abundance, and water quality parameters) and evaluated the functional implications for host-associated (sponge and macroalgae) and free-living (seawater) microbiomes. Coastal inshore reef systems of the Great Barrier Reef (GBR) are characterised by high macroalgal abundance (particularly the canopy-forming brown algae *Sargassum* spp.) and reduced coral cover [36–38]. *Sargassum* biomass on inshore reefs of the GBR fluctuates seasonally and reaches a maximum during early summer and a minimum during mid-winter [36, 39, 40]. Macroalgae-dominated shallow inshore reefs of the GBR are also exposed to larger temperature fluctuations compared with off-shore reefs [41], with sea-surface temperature at inshore reefs frequently reaching 30 °C during summer [42]. Hence, inshore regions of the GBR provide an ideal system to study the effects of macroalgae biomass, temperature, and nutrient fluctuations on the functional potential of coral reef microbiomes.

## Material and methods

### Sample collection and preparation

Marine sponge (*Coscinoderma matthewsi*), macroalgae (*Sargassum* spp.) and seawater samples for metagenomic sequencing were collected during two sampling events (August 2016 and February 2017) at Geoffrey Bay, Magnetic Island (Great Barrier Reef, Queensland, Australia). Additional seawater samples for metagenomic sequencing were collected in June 2016, October 2016, December 2016 and March 2017. Furthermore, seawater (2 L) was collected at all six sampling occasions with a diver-operated Nisikin bottle at 2 m depth for analysis of salinity and quantification of non-purgeable organic carbon (NPOC), non-purgeable inorganic carbon (NPIC), particulate organic carbon (POC), total suspended solids (TSS), chlorophyll *a* (Chl a) and dissolved inorganic nutrients (i.e. ammonium, nitrate, nitrate, phosphate), particulate nitrogen (PN), and total nitrogen (TN) concentrations. Each parameter was measured in duplicate and processed following the standard operational procedures of the Australian Institute of Marine Science (AIMS) [43]. Seawater temperatures specific to the sampling site, date and depth were obtained from AIMS long-term monitoring temperature records (http://eatlas.org.au). Samples were collected under the permit G16/38348.1 issued by the Great Barrier Reef Marine Park Authority.

Samples (*n* = 3 per sample type per sampling event) for metagenome sequencing were collected and processed following the standard operating procedures of the Australian Marine Microbial Biodiversity Initiative (AMMBI) as previously described [44]. In brief, seawater was collected with collapsible sterile bags at 2 m depth and pre-filtered (50 µm) to remove larger particles and subsequently filtered (2 L) onto 0.2 µm Sterivex-filters (Millipore). The sponge *Coscinoderma matthewsi* was removed from the substrate (at 7 m depth) with sterile scalpel blades, rinsed with 0.2 µm filter-sterilised seawater to remove loosely attached microbes from the sponge’s tissue and subsampled into 2 mL cryogenic vials. *Sargassum* spp. was sampled with sterile scalpels at 3 m depth, rinsed with 0.2 µm filtered-sterilised seawater to remove seawater-associated microbes and placed into 2 mL cryogenic vials. All samples were immediately snap frozen in liquid nitrogen and stored at −80 °C.

Prior to DNA extraction, the macroalgal biofilm was separated from the macroalgal tissue [44]. In brief, the biofilm was removed from the macroalgal surface by overnight incubation at 200 rpm in 10 mL 1 × PBS at 37 °C. The suspended biofilm in the supernatant was transferred to a clean tube, centrifuged for 10 min at 16,000 rcf at 4 °C and the resulting pellet was used for DNA extraction. Microbes within the sponge tissue were separated from sponge host cells as described in detail by Botte et al. [45]. Briefly, sponge tissue was rinsed twice (5 min at 200 rpm on an orbital incubation shaker) with sterile calcium- and magnesium-free seawater (CMFSW) and homogenised using a handheld tissue homogeniser (Heidolph Silent Crusher M) for 10 min at 7000 rpm in CMFSW. Next, filter-sterilised collagenase (Sigma Aldrich) was added to the homogenised sponge tissue (final concentration of 0.5 mg/mL) and the tissue slurry incubated on ice for 30 min at 150 rpm on an incubation orbital shaker. After incubation, the microbial cells from the sponge tissue slurry were enriched by a series of filtration and centrifugation steps. The final microbial pellet was recovered in 1 mL Tris-HCl/NaCl and stored at −20 °C until required for DNA extraction.

### DNA extractions and metagenome sequencing

DNA from seawater and macroalgal biofilms was extracted with the DNeasy PowerSoil kit (QIAGEN). DNA of sponge-associated microbial cells was extracted with the Dneasy PowerBiofilm kit (QIAGEN) following the manufacturer’s instructions. DNA extracts were stored at −80 °C until shipment on dry ice to the Australian Genome Research Facility (AGRF; Melbourne, Australia) for sequencing. Libraries were prepared with the Nextera XT Library Preparation Kit (Illumina), following the manufacturer’s protocol and sequenced on a HiSeq 2500 in rapid run mode with 250 bp paired-end reads (24 samples per flow cell resulting in ~5–6 Gbp per sample). Raw sequencing data and metadata are freely available at the Bioplatforms Australia data portal under the Australian Microbiome project (https://data.bioplatforms.com/organization/about/australian-microbiome) and have been deposited under the NCBI BioProject PRJNA594068. A full list of sample identifiers is provided in Supplementary Table S1.

### Read assembly, binning and de-replication

Sequence adaptors of raw reads were removed using SeqPurge v2018_04 [46] and adaptor-trimmed reads of samples were assembled individually with metaSpades v3.13.0 [47] using default settings. Coverage files for metagenomic binning were calculated by mapping adaptor-trimmed reads to assembled scaffolds using BamM v1.7.3 (https://github.com/Ecogenomics/BamM) and metagenome-assembled-genomes (MAGs) were generated with uniteM v0.0.15 (https://github.com/dparks1134/UniteM) using the following binning tools: GroopM v0.3.4 [33], MaxBin v2.2.4 [35] and MetaBAT v2.12.1 [34]. The quality (completeness and contamination) of the resulting MAGs was assessed with CheckM v1.0.12 [48]. The total recovery of MAGs with qualities ≥50 (where quality = completeness−3 × contamination) was estimated with singleM v0.12.1 (https://github.com/wwood/singlem), which quantifies single-copy marker genes in the adaptor-trimmed reads and calculates the percentage of those markers recovered in the MAGs. The total number of bins recovered from sponge, macroalgae and seawater samples, along with bin completeness, contamination and recovery is summarised in Supplementary Table S2. Furthermore, to calculate relative abundances, MAGs from each habitat (sponge, macroalgae, seawater) were first de-replicated separately at 95% Average Nucleotide Identity (95% ANI) using dRep v1.0.0 to avoid arbitrary placement of reads between very similar MAGs [49]. Secondly, adaptor-trimmed reads from samples collected in August 2016 and February 2017 were mapped (75% minimum alignment and 95% minimum identity) against the de-replicated MAGs_95%ANI_ with coverM v0.2.0 (https://github.com/wwood/CoverM). De-replicated MAGs_95%ANI_ have been deposited under NCBI BioProject PRJNA594068.

### Taxonomic assignment and functional annotation of MAGs

Taxonomy was assigned to the MAGs_95%ANI_ using GTDBtk v0.2.1 (https://github.com/Ecogenomics/GTDBTk, see Supplementary Table S3) and functional annotations were assigned with enrichM v0.4.7 (https://github.com/geronimp/enrichM) using the Kyoto Encyclopaedia of Genes and Genomes Orthology (KEGG; KOs). KEGG defines “modules,” which are collections of KOs that together make up a metabolic pathway (e.g. glycolysis) or functional unit (e.g. flagellar assembly). The completeness of KEGG Modules in the individual MAGs_95%ANI_ was assessed using the classify function of enrichM v0.4.7 (https://github.com/geronimp/enrichM) and only KEGG modules with ≥70% completeness were kept in the analysis. Furthermore, representativeness of the retrieved MAGs_95%ANI_ was verified by comparing the taxonomic composition of MAGs_95%ANI_ with metagenomic community profiles generated by extracting 16S rRNA gene fragments of adaptor-trimmed metagenome reads using GraftM v0.12.0 (https://github.com/geronimp/graftM).

Seawater MAGs_95%ANI_ belonging to the phylum Bacteroidota were further screened for the presence of Polysaccharide Utilisation Loci (PULs). To identify the presence of PULs, Bacteroidota MAGs_95%ANI_ were annotated with enrichM v0.4.7 using the Carbohydrate Active enzyme (CAZy) database and the Protein Family (Pfam) database to screen for glycoside hydrolase families (GH) and SusD-like genes (PF07980, PF12741, PF14322 and PF12771), respectively [50].

### Statistical analysis

All statistical analysis was performed in R [51] using the following packages: vegan [52], VennDiagram [53], DESeq [54] and phyloseq [55]. Graphs were created in R using ggplot2 [56] and illustrations were created in Adobe Illustrator.

Variations in the functional profiles of sponge, macroalgae and seawater-associated MAGs_95%ANI_ (presence/absence of KEGG Modules) were evaluated using multivariate statistical approaches including Permutation Multivariate Analysis of Variance (PERMANOVA) and Non-metric multidimensional scaling (NMDS). Dissimilarity matrices of functional presence/absence profiles were generated using the binary Bray Curtis Dissimilarity Index. The number of unique and shared KEGG Modules associated with carbohydrate metabolism, energy metabolism and environmental information processing among sponge, macroalgae and seawater MAGs_95%ANI_ were evaluated using Venn diagrams.

Environmental metadata were z-score standardised [57] and differences between seasons (summer vs. winter) were assessed using *t*-tests. Furthermore, microbial taxa showing significantly different relative abundances between August (peak of winter season) and February (peak of summer season) in sponge, macroalgae and seawater samples, respectively, were evaluated using differential abundance analysis in DESeq. The number of reads mapped to MAGs_95%ANI_ was determined at the phylum level (class for Proteobacteria) for each sample and normalised using variance stabilisation implemented in the DESeq package.

Differences in the functional profiles of microbial taxa that varied significantly between August and February or remained stable between sampling events were further assessed using PERMANOVAs and NMDSs based on binary Bray Curtis Dissimilarities. Similarity Percentage (SIMPER with 10,000 permutations) analysis was used to further pinpoint which KEGG Modules significantly contributed to the observed dissimilarities between August vs. February enriched taxa of macroalgae and seawater microbiomes, and between winter-enriched vs. stable taxa of the sponge microbiome. Log2 fold change of significant KEGG Modules was calculated to compare the proportional changes between groups using the gtools package v3.8.1. in R. In addition, the phylum (class for Proteobacteria) contributing most to the observed change was assessed.

The relative abundance of seawater MAGs_95%ANI_ and environmental metadata were further analysed using Bray–Curtis distance-based redundancy analysis (dbRDA). The best model was selected using the ordistep() function of the vegan package [52] and only significant variables (based on anova.cca() function, *p* < 0.05) were kept in the dbRDA analysis. The explanatory value (in %) of significant environmental variables (i.e. temperature, salinity, silica, total suspended solids and particulate organic carbon) was assessed with a variation partitioning analysis and co-linear environmental parameters (co-linearity threshold of >0.7 or <−0.7) were identified using Pearson correlation. Correlations between the relative abundance of individual seawater MAGs_95%ANI_ and individual environmental variables were analysed using Spearman’s rank correlation.

## Results

### Functional-repertoire of dominant microbes inhabiting macroalgae-dominated inshore reefs

**Fig. 1:**
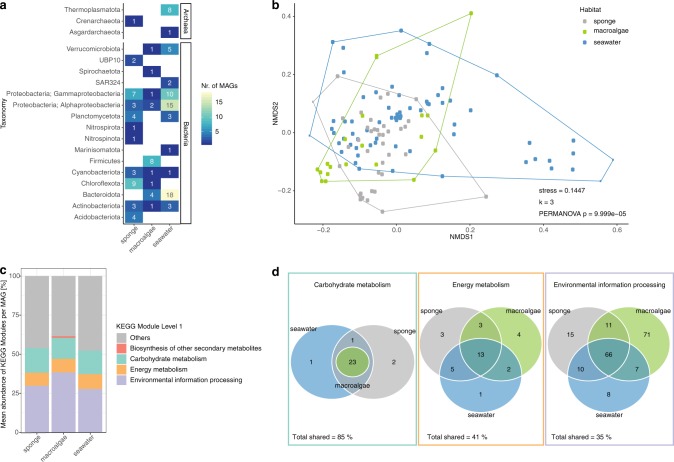
Functional diversity of metagenome-assembled genomes based on 95% average nucleotide identity (MAGs_95%ANI_) associated with sponge tissue, macroalgae biofilms and seawater. **a** Total number of MAGs_95%ANI_ discovered within each bacterial and archaeal phylum (class for Proteobacteria). **b** Non-metric multidimensional scaling plot (NMDS) based on binary Bray Curtis similarity index displaying the functional variability between sponge, algae and seawater MAGs. **c** The average composition of KEGG Modules associated with MAGs for each habitat. The three most abundant categories are shown individually as well as the unique category “Biosynthesis of other secondary metabolites” associated with macroalgae MAGs. The other eight categories are summarised as “Others”. **d** The absolute number of unique and shared KEGG Modules between sponge, macroalgae and seawater MAGs_95%ANI_ for carbohydrate metabolism, energy metabolism and environmental information processing (from left to right).

A total of 125 MAGs_95%ANI_ were recovered, belonging to 15 bacterial and 3 archaeal phyla (Fig. 1a and Supplementary Table S3). Seawater samples yielded the highest number of recovered microbial genomes with 67 MAGs_95%ANI_ (Supplementary Fig. S1), followed by the sponge tissue with 38 MAGs_95%ANI_ (Supplementary Fig. S2) and the macroalgae biofilm with 20 MAGs_95%ANI_ (Supplementary Fig. S3). Recovery of MAGs (calculated with SingleM) varied between habitats, with high-quality MAGs representing 63.5, 27.7 and 35.2% of the total sponge, macroalgae and seawater microbiomes, respectively (Supplementary Table S2). Comparison of taxonomic profiles of MAGs_95%ANI_ with metagenome-derived 16S rRNA gene fragments confirmed that the recovered MAGs_95%ANI_ were representative of the most dominant taxa (Supplementary Fig. S4). Functional profiles of the recovered microbial genomes varied significantly between reef habitats (PERMANOVA F_(2/122)_ = 5.24, *p* = 9.99 × 10^−5^, 10,000 permutations, Fig. 1b). Similar differences in taxonomic and functional diversity were observed when metagenome reads were analysed using a gene-centric approach (Supplementary Fig. S5).

KEGG Modules involved in carbohydrate metabolism, energy metabolism and processing of environmental information represented on average more than half of all KEGG Modules annotated in sponge, macroalgae and seawater MAGs_95%ANI_ (Fig. 1c)_._ The relative abundance of KEGG Modules associated with these three categories was highly similar between habitats (Fig. 1c). The functional category “biosynthesis of other secondary metabolites” was only found in macroalgae MAGs_95%ANI_ (Fig. 1c) and more specifically, referred to the ability of Firmicutes MAGs_95%ANI_ to biosynthesise bacilysin (Supplementary Fig. S6).

To further explore functional similarities between sponge, macroalgae and seawater MAGs_95%ANI_, the number of shared and unique KEGG Modules of the three main categories (carbohydrate metabolism, energy metabolism and environmental information processing processes) of each habitat was enumerated (Fig. 1d and Supplementary Fig. S6). In total, 85% of annotated KEGG Modules relating to carbohydrate metabolism were shared between sponge, macroalgae and seawater MAGs_95%ANI_ (Fig. 1d and Supplementary Fig. S6). KEGG Modules of the central carbohydrate metabolism (i.e. glycolysis, pentose phosphate pathway, Entner–Doudoroff pathway, citrate cycle) showed 100% overlap between habitats (Supplementary Fig. S6). In contrast, only 41 and 35% of KEGG Modules related to energy metabolism and environmental information processing, respectively, were shared between sponge, macroalgae and seawater MAGs_95%ANI_ (Fig. 1d and Supplementary Fig. S6)_._ Carbon fixation (such as Calvin cycle and Arnon–Buchanan cycle, also referred to as reductive citric acid cycle) and ATP synthesis KEGG Modules were highly conserved between habitats (Supplementary Fig. S6). A higher variability between habitats was observed in the potential to metabolise methane (i.e. formaldehyde assimilation and methane oxidation), nitrogen (i.e. assimilatory and dissimilatory nitrate reduction) and sulphur (i.e. assimilatory and dissimilatory sulphate reduction, and sulphate oxidation) as well as the potential to gain energy through photosynthesis (Supplementary Fig. S6). The highest number of unique environmental information processing KEGG Modules was observed in the algae MAGs_95%ANI_ (Fig. 1d). These unique KEGG Modules are mainly involved in antibiotic resistance and antibiotic transport, the transfer of sugar molecules via phosphorylation (phosphotransferase system) and two-component regulatory systems for chemosensory, virulence and antibiotic biosynthesis (Supplementary Fig. S6). Environmental information processing KEGG Modules exclusively associated with sponge MAGs_95%ANI_ (Fig. 1e and Supplementary Fig. S6) included copper-processing transport system, antibiotic transport and resistance, cationic antimicrobial peptide (CAMP) resistance, type IV secretion systems and two-component regulatory systems (i.e. nitrogen fixation, nitrate respiration, metal and copper tolerance, and quorum sensing). KEGG Modules exclusively associated with seawater MAGs_95%ANI_ included transporters for Glycerol and N-Acetylglucosamine and two-component regulatory systems for glutamine utilisation, C4-dicarboxylate transport, type four fimbriae synthesis, and tricarboxylic acid transport. KEGG Modules ubiquitously present in seawater, sponge and macroalgae MAGs_95%ANI_ were the ABC-2 type transport systems, aminoacyl tRNA metabolism, twin-arginine translocation (Tat) system, Sec (secretion) system, phosphate transport system as well as a phosphate starvation response two-component regulatory system.

### Seasonal variation in environmental conditions

Seawater temperature significantly changed (*t*-test; *p* = 0.0039) between winter and summer at the sampling location (Supplementary Fig. S7) and ranged between 23 °C in August and 30 °C in February (Supplementary Fig. S8). Ammonium (NH_4_^+^) concentration and nitrite plus nitrate (NO_2_^−^:NO_3_^−^) concentration were positively correlated with increasing seawater temperature (Pearson correlation >0.7; Supplementary Fig. S9). In contrast, total suspended solids (TSS) were negatively correlated with seawater temperature (Pearson correlation <−0.7; Supplementary Fig. S9). Furthermore, *Sargassum* spp. abundance was highest during summer (personal observation) and followed previously described seasonal growth-decay patterns typical for this sampling site and for inshore reefs of this region [36, 40]. Macroalgae cover at the sampling site has been reported to reach up to 54.8% [36].

### Shifts in microbial taxa alter the functional potential of reef microbiomes

**Fig. 2:**
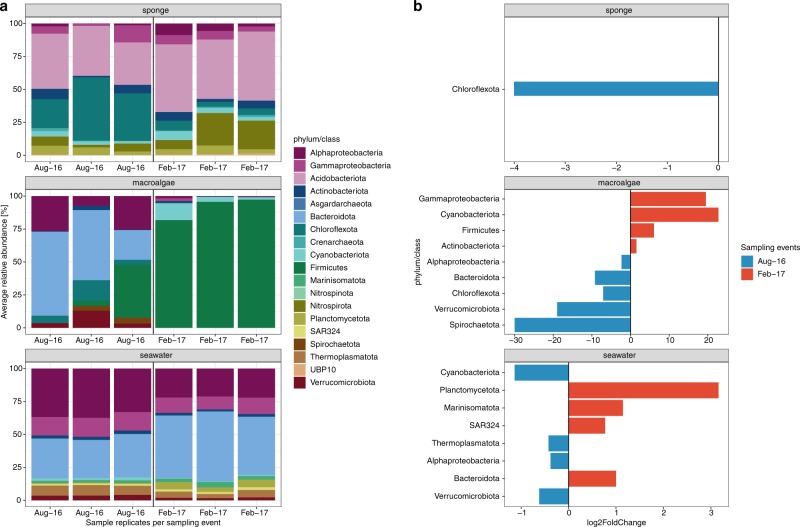
Compositional stability of microbiomes associated with sponge tissue, macroalgae biofilms and seawater between winter (August) and summer (February). **a** Relative abundances of metagenome-assembled genomes based on 95% average nucleotide identity (MAGs_95%ANI_) on phylum (class for Proteobacteria) level in the sample replicates collected in August 2016 and February 2017. **b** Log2 fold change of significantly enriched microbial phyla (class for Proteobacteria) between winter and summer sampling events based on differential abundance analysis (DESeq).

**Fig. 3:**
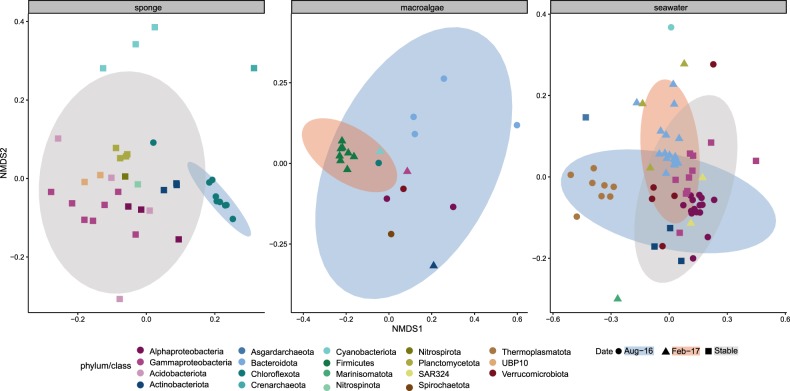
Functional profiles of metagenome-assembled genomes based on 95% average nucleotide identity (MAGs_95%ANI_) associated with sponge tissue, macroalgae biofilm and seawater. Non-Metric Dimensional Scaling plot based on binary Bray Curtis Dissimilarities displaying variations in the functional profiles (KEGG Module presence/absence) between MAGs_95%ANI._ Colour represents phylum of MAGs_95%ANI_ (class for Proteobacteria) and shape represent whether a phylum (class for Proteobacteria) was significantly enriched during a sampling time point (August vs. February) or stable between sampling time points (Stable). Hulls represent the multivariate t-distribution of groups (August, February, Stable).

**Fig. 4:**
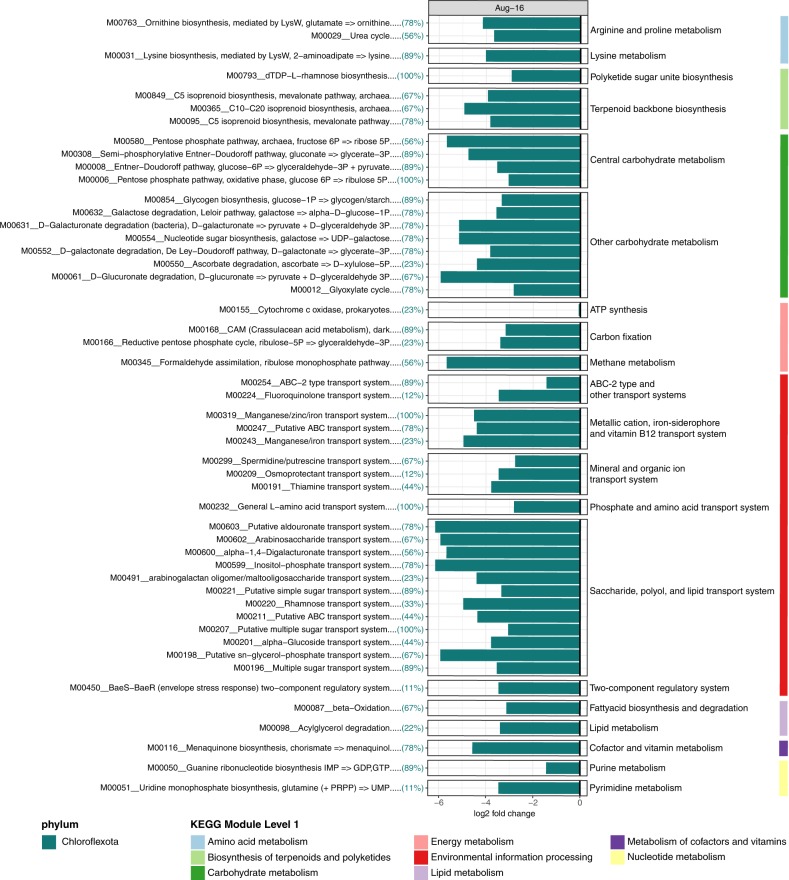
Sponge-associated microbial functions were significantly associated with the winter-enriched phylum Chloroflexota. KEGG Modules significantly (*p* < 0.05) driving the observed functional dissimilarities of enrichment groups (August vs. Stable) were evaluated with Similarity Percentages (SIMPER). The enrichment of significant KEGG Modules is displayed as log2 fold change. Colour of the bar chart indicates the microbial taxa contributing most to the observed function and the number of Chloroflexota MAGs_95%ANI_ (in percent) harbouring the individual KEGG Modules is provided between brackets.

Sponge-affiliated microbial taxa remained highly stable between winter and summer sampling events (Fig. 2a and Supplementary Fig. S2), with only 1 of 11 taxa varying significantly (based on differential relative abundance analysis using DESeq). The phylum Chloroflexota (9 MAGs_95%ANI_) was significantly enriched in winter samples and reduced by 85% in summer (Fig. 2b and Supplementary Table S4a). In conjunction, functional profiles of the phylum Chloroflexota differed significantly from the stable microbial community, comprised of microbial taxa that remained equally abundant between sampling time points (PERMANOVA F_(1/36)_ = 9.56, *p* = 9.99 × 10^−5^, 10,000 permutations; Fig. 3). KEGG Modules driving the significant functional dissimilarity between winter and stable summer microbial taxa (based on SIMPER) were predominantly affiliated with Chloroflexota MAGs_95%ANI_ (Fig. 4). A substantial reduction in Chloroflexota within the sponge could have implications for the microbiome’s ability to metabolise carbohydrates such as glucose and fructose, and for the ability to transfer sugar molecules between the microbiome and the host (decrease in saccharide transport systems; see Fig. 4). In addition, the pentose phosphate shunt, a glucose oxidation pathway, was significantly linked with Chloroflexota MAGs_95%ANI_. Other KEGG Modules significantly affiliated with Chloroflexota MAGs_95%ANI_ were vitamin B1 (thiamine) transport system, antibiotic transport systems (fluoroquinolone) and metal transport systems (e.g. manganese, zinc and iron).

**Fig. 5:**
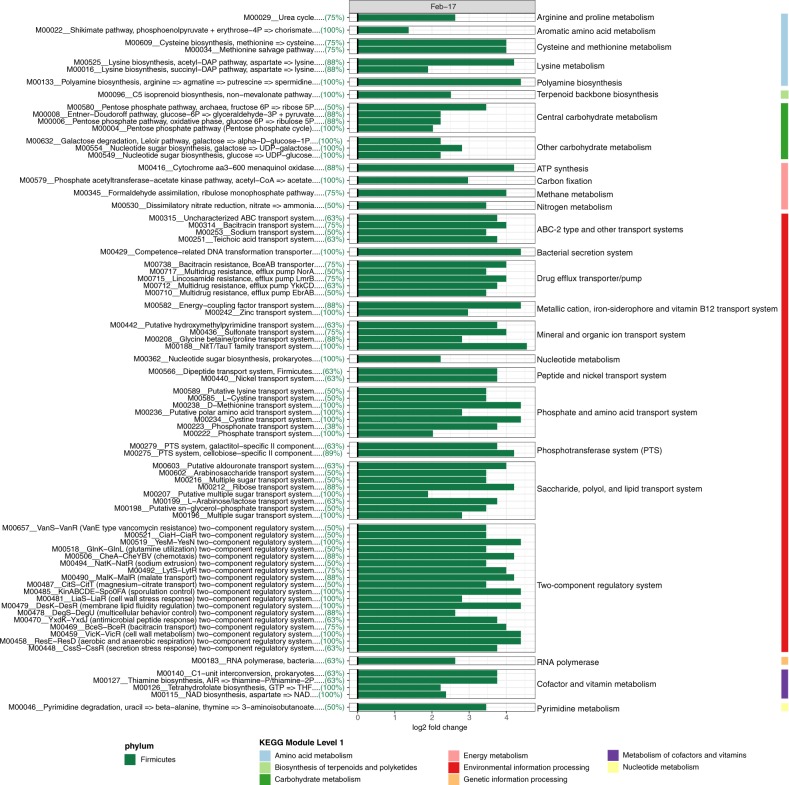
Macroalgae associated microbial functions were significantly associated with summer-enriched taxa. KEGG Modules significantly (*p* < 0.05) driving the observed functional dissimilarities of enrichment groups (August vs. February) were evaluated with Similarity Percentages (SIMPER). The enrichment of significant KEGG Modules is displayed as log2 fold change. Colour of the bar chart indicates the microbial taxa contributing most to the observed function and the number of Firmicutes MAGs_95%ANI_ (in percent) harbouring the individual KEGG Modules is provided between brackets.

In contrast to the sponge microbiome, macroalgae biofilm MAGs_95%ANI_ varied significantly between sampling events (Fig. 2a and Supplementary Fig. S3), with all 9 microbial taxa significantly enriched in either winter or summer samples (Fig. 2b; based on differential abundance analysis using DESeq). During winter the phyla Spirochaetota (1 MAG_95%ANI_), Verrucomicrobiota (1 MAG_95%ANI_), Bacteroidota (4 MAGs_95%ANI_), Chloroflexota (1 MAG_95%ANI_) and the class Alphaproteobacteria (2 MAGs_95%ANI_) were significantly enriched, whereas the phyla Actinobacteriota (1 MAG_95%ANI_), Firmicutes (8 MAGs_95%ANI_), Cyanobacteria (1 MAG_95%ANI_) and the class Gammaproteobacteria (1 MAG_95%ANI_) were significantly enriched during summer (Fig. 2b and Supplementary Table S4b). The significant variation in microbial taxa within the macroalgae biofilm also had implications for the underlying functional repertoire (February vs. August, PERMANOVA F_(1/18)_ = 4.92, *p* = 9.99 × 10^−5^, 10,000 permutations; Fig. 3). Firmicutes, for example, were significantly enriched during summer (Fig. 2b) and were the primary contributors to the observed functional dissimilarities between sampling time points (Fig. 5). KEGG Modules associated with Firmicutes MAGs_95%ANI_ included degradation of carbohydrates (i.e. galactose and glucose) and the uptake of carbohydrates upon phosphorylation (phosphotransferase system - PTS). The observed PTSs were specific to galactitol and cellobiose. Saccharide and polyol transport systems were also enriched in Firmicutes MAGs_95%ANI_ (Fig. 5). Furthermore, dissimilatory nitrate reduction (nitrate respiration) and a two-component regulatory system activating aerobic and anaerobic respiration genes were found in summer-enriched Firmicutes MAGs_95%ANI_. Firmicutes-dominated biofilms were also enriched in KEGG Modules encoding for antibiotic and multidrug resistance and transport systems as well as two-component regulatory systems for antibiotic resistance, behaviour control, sporulation control and stress response (Fig. 5).

**Fig. 6:**
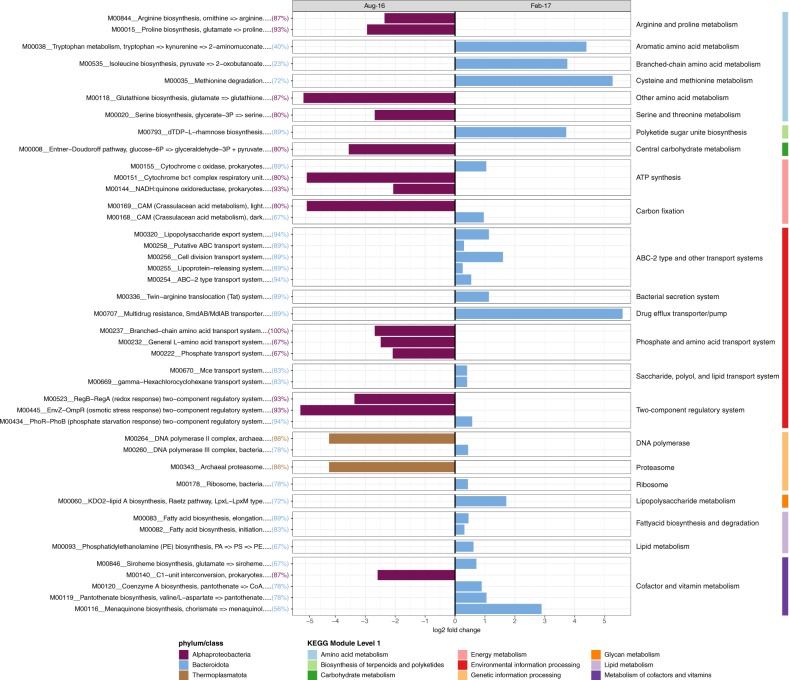
Seawater microbial functions were significantly associated with winter and summer-enriched taxa. KEGG Modules significantly (*p* < 0.05) driving the observed functional dissimilarities of enrichment groups (August vs. February) were evaluated with Similarity Percentages (SIMPER). The enrichment of significant KEGG Modules is displayed as log2 fold change. Colour of the bar chart the microbial taxa contributing most to the observed function and the number of Alphaproteobacteria, Thermoplasmatota, and Bacteroidota MAGs_95%ANI_ (in percent) harbouring the individual KEGG Modules is provided between brackets.

Seawater samples, like the macroalgae biofilm, displayed high variability in abundant members between sampling events, with 8 of 11 microbial taxa varying significantly (based on differential relative abundance analysis using DESeq) between winter and summer sampling events (Fig. 2 and Supplementary Fig. S1). The bacterial phyla Bacteroidota (18 MAGs_95%ANI_), SAR324 (2 MAGs_95%ANI_), Marinisomatota (1 MAG_95%ANI_), Plancotmycetota (3 MAGs_95%ANI_) were significantly enriched during summer, while the bacterial phyla Verrucomicrobiota (5 MAGs_95%ANI_), Cyanobacteriota (1 MAGs_95%ANI_), Proteobacteria (class Alphaproteobacteria; 15 MAGs_95%ANI_) and the archaeal phylum Thermoplasmatota (8 MAGs_95%ANI_) were significantly enriched in winter (Fig. 2b and Supplementary Table S4c). Seawater temperature (and co-varying ammonium, NH_4_^+^, and nitrite plus nitrate, NO_2_^−^:NO_3_^−^), total suspended solid concentration (TSS), non-purgeable organic carbon (NPOC) concentration, and silica (SiO_2_) concentration in the seawater explained 96.6% of the observed variation in the MAGs_95%ANI_ community profiles (Variation Partitioning Analysis; Supplementary Fig. S10). Seawater microbial taxa enriched in winter, summer, and the stable microbial community (August vs. February vs. Stable) also exhibited significant differences in their functional profiles (PERMANOVA F_(2/64)_ = 4.79, *p* = 9.99 × 10^−5^, 10,000 permutations; Fig. 3). These results suggest that seasonal shifts of microbial taxa (Fig. 2) lead to significant changes in the functional potential of pelagic reef microbiomes (Fig. 3). Functional dissimilarities between sampling time points were mainly attributed to winter-enriched Alphaproteobacteria MAGs_95%ANI_ and archaeal Thermoplasmatota MAGs_95%ANI_, and the summer-enriched Bacteroidota MAGs_95%ANI_ (Fig. 6). Furthermore, the relative abundance of Bacteroidota MAGs_95%ANI_ was positively correlated with an increase in sea-surface temperature, ammonium (NH_4_^+^), phosphate (PO_4_^3-^), nitrite (NO_2_^−^), nitrite plus nitrate (NO_2_^−^:NO_3_^−^), and silica (SiO_2_) concentration in the seawater (Supplementary Fig. S11). In contrast, alphaproteobacterial MAGs_95%ANI_ of the order Rhodobacterales and Pelagibacterales, as well as MAGs_95%ANI_ of the phylum Thermoplasmatota were positively correlated with total suspended solids (TSS), salinity, and non-purgeable organic carbon (NPOC; Supplementary Fig. S11). The increase of Bacteroidota MAGs_95%ANI_ led to an increase in KEGG Modules potentially linked to virulence and pathogens. For example, Bacteroidota MAGs_95%ANI_ were enriched in the biosynthesis of polyketide sugars (dTDP-L-rhamnose biosynthesis), KDO2-lipid A biosynthesis (Raetz pathway), lipoprotein releasing system and the twin-arginine translocation (Tat) system. Furthermore, Bacteroidota MAGs_95%ANI_ showed genomic potential for phosphatidylethanolamine (PE) biosynthesis. Aromatic amino acid metabolism (tryptophan metabolism) and methionine degradation (sulphur-containing amino acid) were also enriched during summer. In addition, Bacteroidota MAGs_95%ANI_ were all equipped with SusD-like genes and glycoside hydrolase (GH) families (Supplementary Tables S5 and S6), indicating their ability to degrade polysaccharides via the PUL machinery. The GH 16 family was the most abundant GH in the Bacteroidota MAGs_95%ANI_.

## Discussion

The contribution of microbes to the health of reef holobionts has been the focus of much recent research [9, 11, 58], and microbial involvement in the perpetuating cycle of reef degradation via the DDAM feedback loop further highlights the central role of microorganisms in coral reef ecosystem health and resilience [8, 28]. However, the function of individual microorganisms across different coral reef habitats and across seasons remains poorly understood. Here we identify the functional potential of different members of sponge, macroalgae and seawater microbiomes using genome-centric metagenomics. We show that shifts in the relative abundance of individual taxonomic groups between winter and summer can have implications for the functional potential of a microbiome and hence, the stability of reef holobionts and ecosystems.

Planktonic Bacteroidota MAGs_95%ANI_ (i.e. family Flavobacteriaceae and Cryomorphaceae, see Supplementary Table S3) were significantly enriched during summer, corresponding with peaks in sea-surface temperature, ammonium (NH_4_^+^) concentration, nitrite plus nitrate (NO_2_^−^:NO_3_^−^) concentrations (Supplementary Figs. S7–S9), and the abundance of canopy-forming brown algae on inshore reefs of the GBR [36, 40, 42]. Furthermore, the relative abundance of individual planktonic Bacteroidota MAGs_95%ANI_ was positively correlated with sea-surface temperature and nutrient concentration (i.e. ammonium, phosphate, nitrate, nitrite:nitrate, and silica concentrations) in the seawater (Supplementary Fig. S11). Recent 16S rRNA gene-based studies have reported similar increases in Flavobacteriaceae and Cryomorphaceae in macroalgae-dominated reefs [8], and in inshore-reefs of the GBR, particularly when sea-surface temperatures are high [44]. Marine Bacteroidota are known to degrade macroalgae-derived polysaccharides via a unique machinery referred to as polysaccharide utilisation loci (PULs, [59]) and are major responders to phytoplankton blooms in temperate waters [50]. The summer-enriched Bacteroidota MAGs_95%ANI_ were all equipped with glycoside hydrolase (GH) families and SusD-like genes (Supplementary Tables S5 and S6), revealing their genomic potential to degrade a diverse range of polysaccharides via the PUL machinery. The most abundant GH in the Bacteroidota genomes was the GH16 family (Supplementary Table S5), which includes enzymes specific for the degradation of marine polysaccharides [60]. For example, the GH16 contains the enzyme laminarinase which is known to hydrolyse the *β*-1,3-d-linked main chain of laminarin into glucose and oligosaccharides [61]. The ubiquitous presence of genes encoding for the GH16 family in Bacteroidota MAGs_95%ANI_ suggests that planktonic Bacteriodota are capable of degrading macroalgae-derived polyschacharides such as laminarin, a common storage *ß*-glucan of brown algae [62]. Furthermore, the laminarinase enzyme was recently shown to be ubiquitously present in genomes of marine Bacteroidota [50], hence, increased Bacteroidota in the seawater microbiome may be directly linked to increased macroalgal biomass in the reef ecosystem. Interestingly, Bacteroidota were also recently shown to be enriched in coral microbiomes when experimentally exposed to increased macroalgal cover [19]. Elevated seawater temperatures can enhance the exudation of macroalgae-derived polysaccharides [63] which may also be contributing to the summertime enrichment of Bacteroidota. However, experimental validation is required to confirm the direct response of planktonic Bacteroidota to macroalgae proliferation, high seawater temperatures, and nutrient concentrations in coral reef ecosystems.

**Fig. 7:**
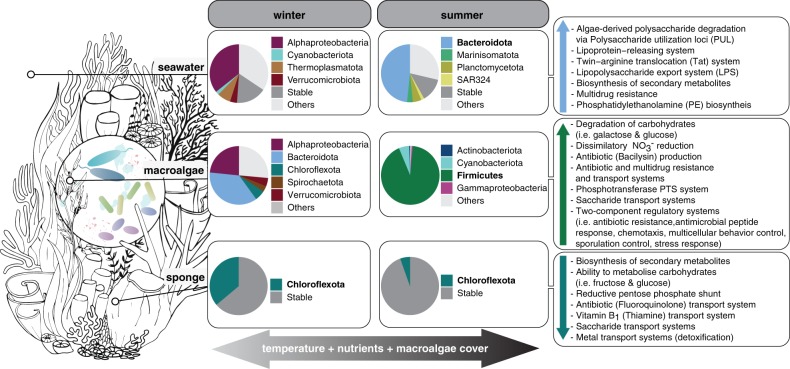
Conceptual overview of seasonal changes in coral reef microbiomes. Elevated seawater temperature, increased macroalgal abundance, and nutrient concentrations during summer are correlated with a shift in the taxonomic composition of seawater, macroalgae and sponge microbiomes and an associated increase in microbial functions associated with opportunistic/pathogenic and copiotrophic process.

In addition to their proposed role in degradation of macroalgae-derived polysaccharides on reefs, planktonic Bacteroidota MAGs_95%ANI_ were also enriched in putative virulence and pathogenic marker genes (Figs. 6 and 7). For example, Bacteroidota MAGs_95%ANI_ have genomic potential to biosynthesise polyketide sugars (dTDP-L-rhamnose biosynthesis) and KDO2-lipid A biosynthesis (Raetz pathway). Polyketide sugars are integrated into the lipopolysaccharide (LPS) layer of gram-negative bacteria and can help pathogens escape host detection [64]. KDO2-lipid A is an essential component of the LPS layer, which can stimulate a host immune response and modulate virulence [65]. Furthermore, the lipoprotein releasing system and the twin-arginine translocation (Tat) system (releasing system of folded-proteins) were enriched in summer-elevated Bacteroidota MAGs_95%ANI_. Lipoproteins play key roles in adhesion to host cells, modulation of inflammatory processes and translocation of virulence factors into host cells and can be released via the Tat system [66]. The genomic potential to biosynthesise phosphatidylethanolamine (PE), an unsaturated fatty acid, was also enriched in Bacteroidota MAGs_95%ANI_. This may allow members of the phylum Bacteroidota to tolerate higher temperatures as the increase of unsaturated fatty acids in the LPS significantly contributes to membrane fluidity [67].

In contrast to Bacteroidota-driven processes during summer, Alphaproteobacteria and Thermoplasmata MAGs_95%ANI_ were enriched in reef waters during winter (Figs. 3, 7, and Supplementary Fig. S11) when sea-surface temperatures and nutrient concentrations are low and macroalgae essentially disappears from inshore GBR reefs [36, 40, 42]. An increase in the relative abundance of the alphaproteobacterial families Pelagibacteraceae and Rhodobacteraceae has previously been described for inshore GBR waters during winter [44, 68] and is also more generally associated with increased coral cover [8, 69]. The archaeal Thermoplasmata (Marine Group II) have previously been reported as abundant members of the planktonic microbial community of the GBR, with increasing relative abundances in off-shore reef locations and in reefs with high coral cover [68].

Collectively, our findings support previous studies reporting increased copiotrophic microorganisms (i.e. Bacteroidota) and virulence factors on reefs with high macroalgal cover [8] and with predictions based on the DDAM feedback loop [28]. Hence, we propose that increased Bacteroidota to Alphaproteobacteria ratios in reef waters may act as an indicator of enhanced macroalgal growth, increased nutrient levels, and the onset of microbialisation in coral reefs. However, in contrast to the previously described shift in central carbohydrate metabolism (i.e. Embden–Meyerhof pathway, Entner–Doudoroff pathway, and pentose phosphate pathway) between coral and macroalgae-dominated reefs [8], the potential to metabolise carbohydrates remained relatively stable between Alphaprotebacteria and Bacteroidota (Supplementary Fig. S12). The only exception being the Entner–Doudoroff pathway which was more prominent in the winter-enriched Alphaproteobacteria compared with summer-enriched Bacteroidota genomes (Fig. 6). Given the importance of microbially mediated carbohydrate metabolism in coral reefs, identifying changes in the central metabolic pathways of Alphaproteobacteria and Bacteroidota using sensitive transcriptome/proteomic approaches is warranted.

*Sargassum* spp., a canopy-forming brown algae, undergoes an annual cycle of growth, reproduction and senescence [70]. In inshore regions of the GBR, *Sargassum* grows rapidly between October and February, followed by a period of senescence during which it sheds most of its fronds [70]. During summer, Firmicutes dominate the macroalgal biofilm (up to 91.4% of the microbiome), having the genomic capacity to generate a hostile environment via production of antibiotics (i.e. Bacilysin) which may hinder opportunistic and biofouling microbes from colonising the macroalgae’s surface (Figs. 2 and 7). Furthermore, Firmicutes MAGs_95%ANI_ had the potential to take up various saccharides (i.e. cellobiose, galactitol, fructose, mannose and mannitol) via the PTS; with the sugar alcohol mannitol being characteristic for *Sargassum* [71]. The PTS is a common feature of Firmicutes and in addition to its primary metabolic function, it is recognised for its regulatory role in biofilm formation, virulence and nitrogen utilisation [72]. In contrast, Bacteroidota predominated in the biofilm during winter (Figs. 2 and 7), when *Sargassum* is reduced to a holdfast with one or two short primary axes [70]. A significant role for seaweed-associated microbes in host morphogenesis has previously been reported [73–75] and the Firmicutes to Bacteroidota ratio may also play a direct role in the growth-decay cycle of *Sargassum*. Based on the observed shift in biofilm-associated microbial taxa between summer and winter, we hypothesise that the *Sargassum* spp. biofilm undergoes a microbial succession synchronised with the seasonal growth-decay cycle of the host and possibly with the availability of sugars. However, high temporal resolution sampling over multiple years would be needed to validate the links between the state of the *Sargassum* spp. biofilm and the annual cycle of growth, reproduction and senescence of the host. In addition, macroalgal surfaces may provide a seed bank for planktonic Bacteroidota thriving on algal-exudates (detection of four seawater Bacteroidota genomes in macroalgae samples; see Supplementary Table S7) as well as potentially antagonist bacterial taxa for corals. For example, macroalgal contact can destabilise the coral microbiome and facilitate growth of many conditionally rare taxa [19]. Firmicutes and Bacteroidota have been shown to significantly increase in corals exposed to short-term stress including elevated temperature and macroalgae-abundance [19, 25, 27, 76]. Hence, understanding the functional roles of Bacteroidota and Firmicutes on coral reefs and assessing their potential to invade carbohydrate-rich niches (e.g. coral mucus) is critical.

Marine sponges are a highly diverse component of coral reefs [77] where they provide a vital trophic link between the benthic and pelagic realms by removing dissolved organic matter from the reef seawater, making it available to benthos-dwelling life forms as particulate material [20]. The health of a sponge holobiont is underpinned by its microbiome [14, 58, 78]. High microbial abundance (HMA) sponges, such as *C. matthewsi* commonly associate with Chloroflexota [79]. The Chloroflexota MAGs_95%ANI_ in this study showed genomic potential to biosynthesise secondary metabolites such as dTDP-L-rhamnose (Fig. 4), a polyketide sugar and O antigen in the bacterial cell wall, which is hypothesised to help sponge amoebocytes differentiate between symbiont and food bacteria [64, 80]. A reduction in sponge-associated Chloroflexota could have adverse consequences for host nutrition (carbohydrate metabolism), B-vitamin availability, detoxification (heavy metal detoxification), waste product removal (urea cycle), and the overall health of the sponge holobiont (Figs. 4 and 7). The observed reduction in Chloroflexota during summer may reflect a shift in substrate availability (e.g. increased ammonium (NH_4_^+^) concentration and nitrite plus nitrate (NO_2_^−^:NO_3_^−^) concentration) and/or a temperature-induced loss of a putative symbiont (Figs. 2 and 7), as has been reported in other sponge species under thermal stress [17]. However, other biological variables (e.g. sponge physiology) not measured in this study could have also contributed to the observed variation in the sponge microbiome.

## Conclusions

Genome-centric metagenomic analysis of host-associated and free-living microbiomes has revealed the functional potential of dominant microbial taxa within an inshore coral reef on the GBR. Further, comparative analysis across seasons allowed to us to identify four bacterial groups (Bacteroidota, Alphaproteobacteria, Firmicutes and Chloroflexota) whose genomic repertoire (Supplementary Figs. S12–S14) and correlation to environmental fluctuations (e.g. seawater temperature, macroalgae abundance and nutrient availability) suggests a key role in coral reef ecosystems processes. We, therefore, propose that future reef research should employ sensitive metatranscriptome/metaproteome and stable isotope-based approaches to (i) validate macroalgae, nutrient, and temperature-related shifts in Bacteroidota to Alphaproteobacteria ratios in reef seawater, (ii) investigate the direct/indirect roles of Firmicutes in the health of reef holobionts and (iii) validate the impacts of environmentally driven fluctuations in symbiont abundance on host health.

## Supplementary information

Supplementary Material
