# Supplementary material for: Comparative genome-centric analysis reveals seasonal variation in the function of coral reef microbiomes
Source: ISME J. 2020 Mar 2;14(6):1435–50. doi: 10.1038/s41396-020-0622-6 (PMC7242418; doi:10.1038/s41396-020-0622-6)
Supplement: Supplementary file 1 — Supplementary Material [file 41396_2020_622_MOESM1_ESM.pdf]

Comparative genome-centric analysis reveals seasonal variation in the function of coral reef microbiomes

Bettina Glasl[a,b,c,*], Steven Robbins[d], Pedro R. Frade[e], Emma Marangon[a,b,c], Patrick Laffy[a], David G. Bourne[a,b,c], and Nicole S. Webster[a,c,d]

[a] Australian Institute of Marine Science, Townsville, Qld, Australia

[b] College of Science and Engineering, James Cook University, Townsville, Qld, Australia

[c] AIMS@JCU, Townsville, Qld, Australia

[d] Australian Centre for Ecogenomics, University of Queensland, Brisbane, Qld, Australia

[e] Centre of Marine Sciences, University of Algarve, Faro, Portugal

* Corresponding author: Bettina Glasl (b.glasl@aims.gov.au)

Content:

- 14 Supplementary Figures
- 7 Supplementary Tables

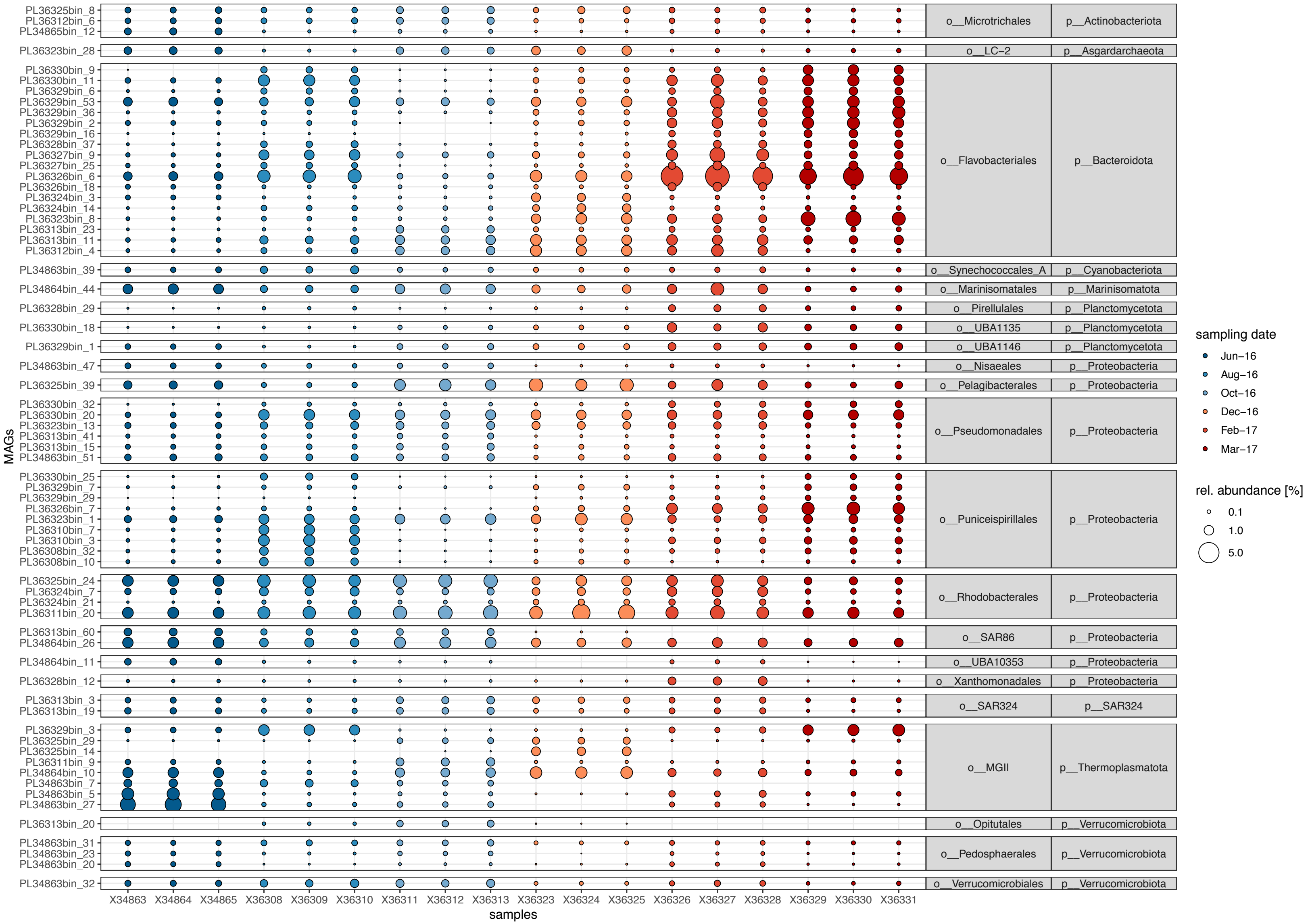

Figure S1. Relative abundance of seawater $MAGs_{95\%ANI}$.

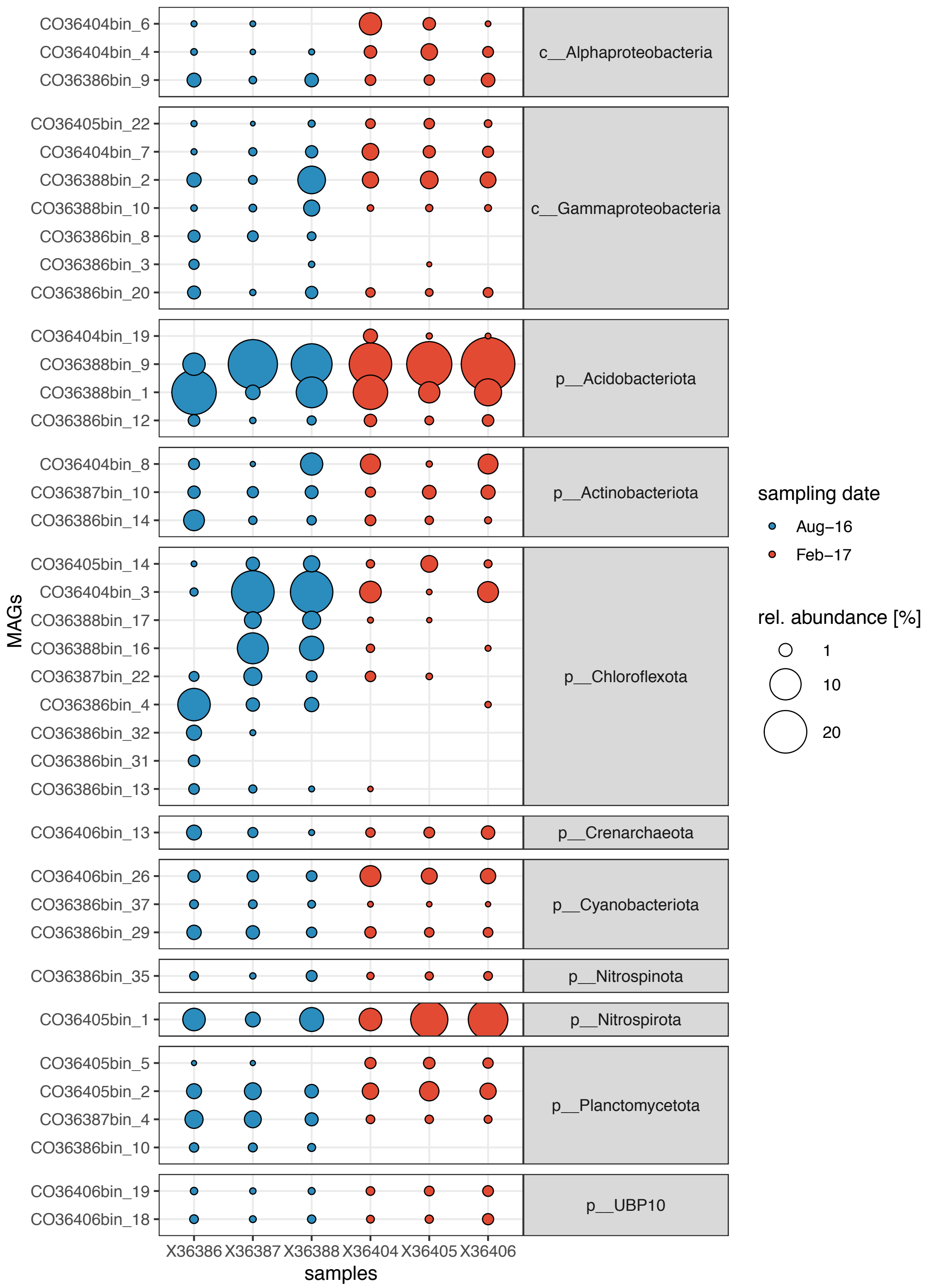

Figure S2. Relative abundance of sponge MAGs$_{95\%ANI}$.

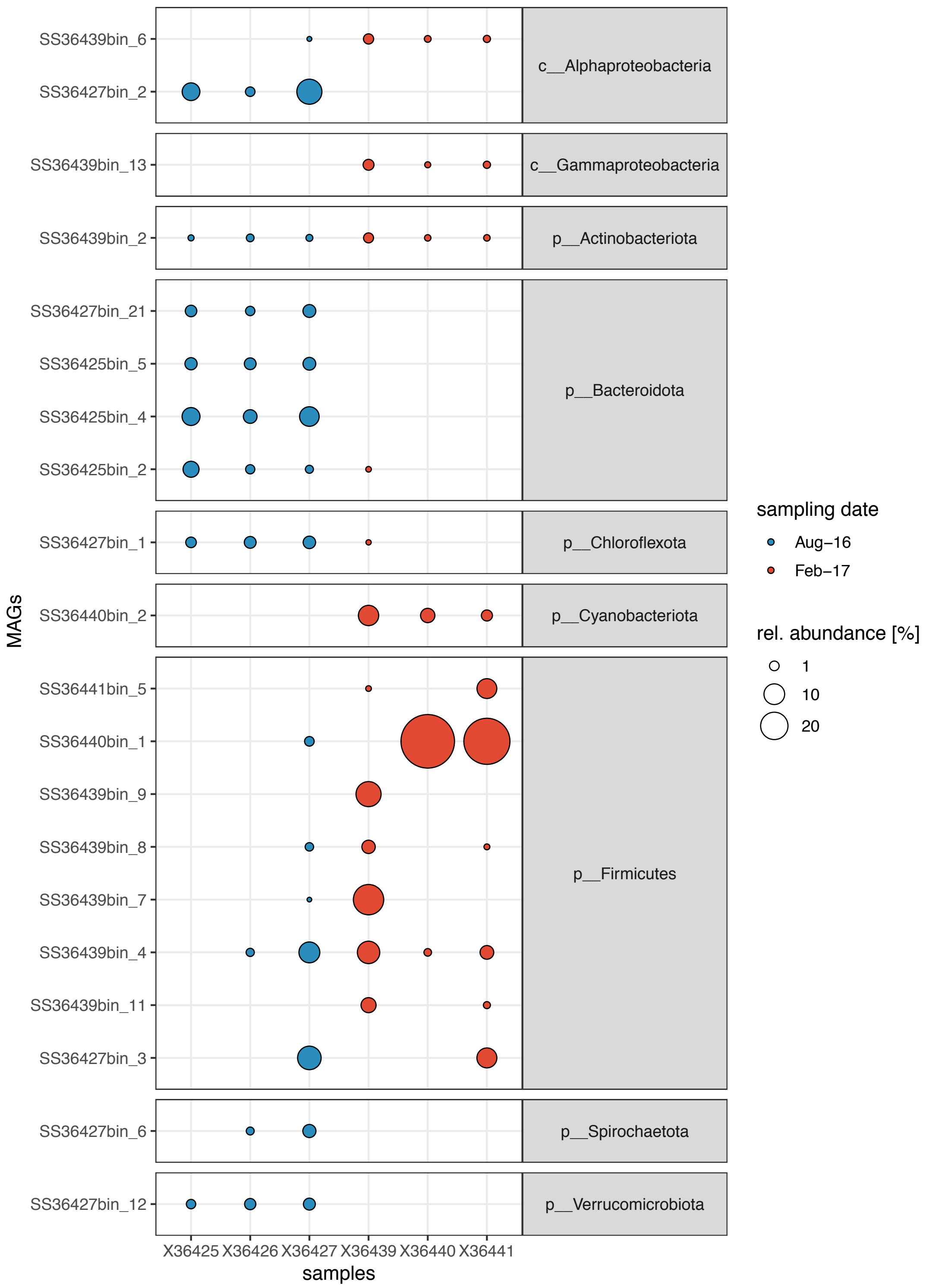

Figure S3. Relative abundance of macroalgae $MAGs_{95\%ANI}$.

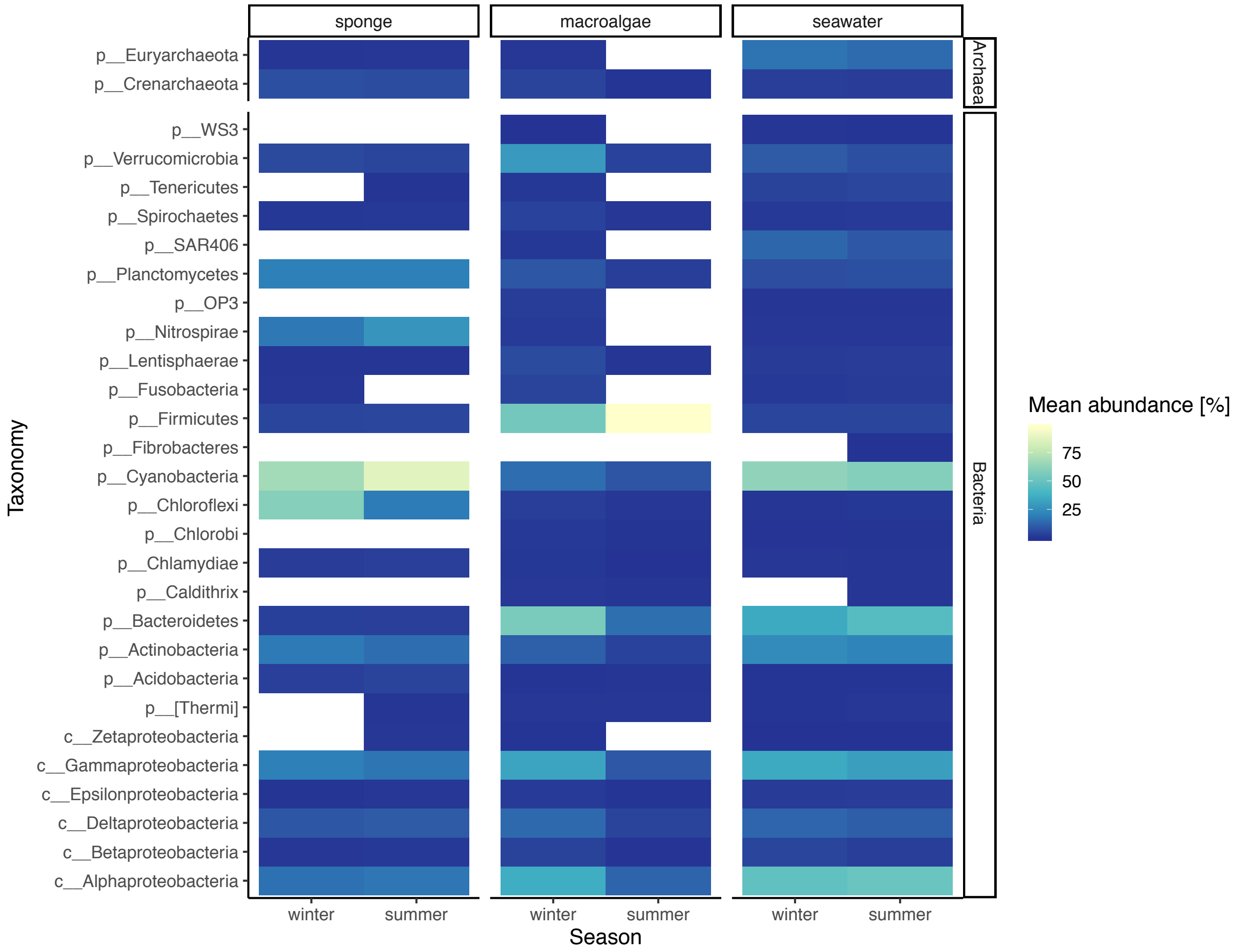

Figure S4. Average relative abundance of microbial taxa based on 16S rRNA genes present in the metagenomic reads in sponge, macroalgae, and seawater samples.

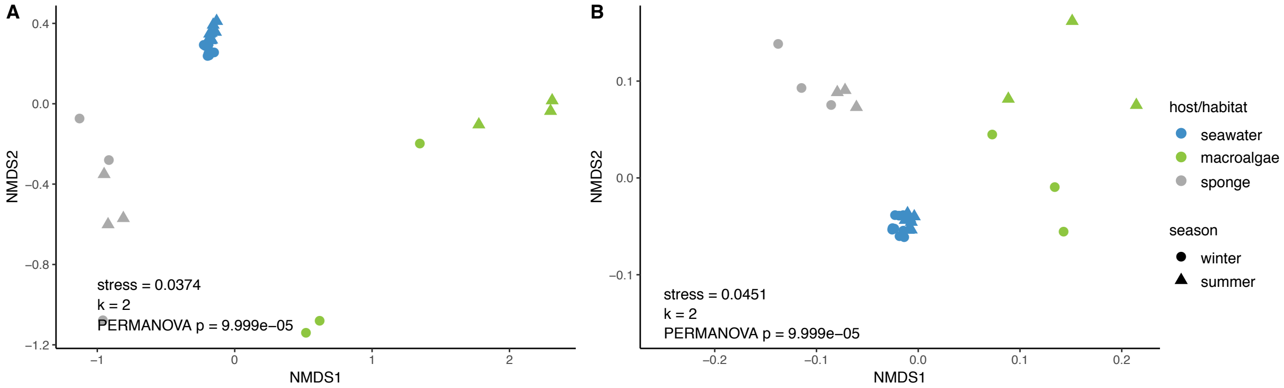

Figure S5. Gene-centric approach: taxonomic and functional annotation of metagenome reads. A) Community composition based on adapter trimmed reads was inferred using GraftM v0.12.0 (https://github.com/geronimp/graftM). B) Assembled reads (scaffolds) were used to assess the functional variability of seawater, sponge and macroalgae microbiomes. The functional annotation was performed with enrichM v0.4.7 (https://github.com/geronimp/enrichM) using the KEGG Orthology (KOs). The functional and taxonomic variability between seawater, sponge and macroalgae microbiomes was assessed with PERMANOVAs and Non-metric multidimensional scaling (NMDS) based on Bray-Curtis dissimilarities in R using the vegan package.

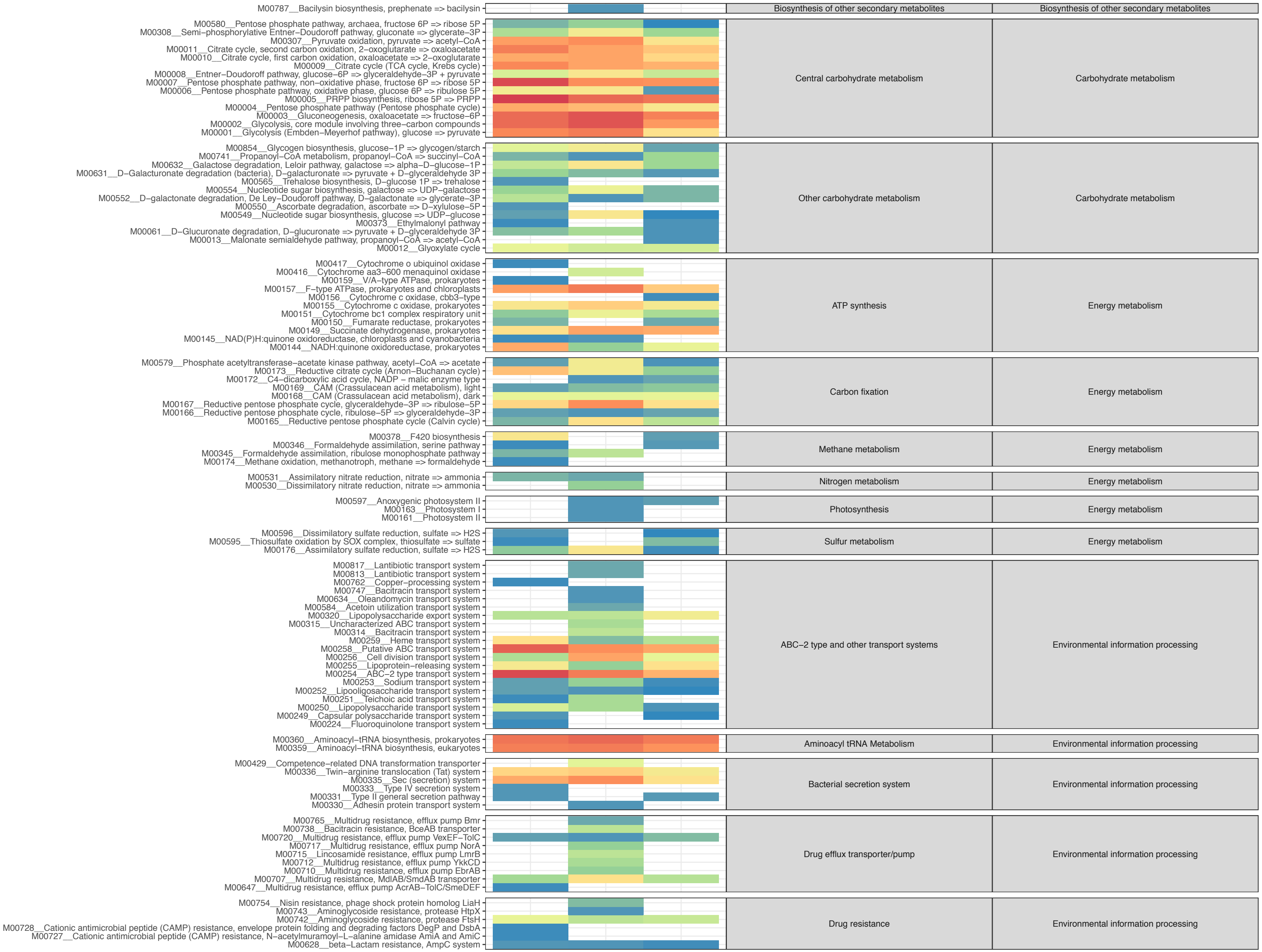

M00787__Bacilysin biosynthesis, prephenate => bacilysin
M00580__Pentose phosphate pathway, archaea, fructose 6P => ribose 5P
M00308__Semi−phosphorylative Entner−Doudoroff pathway, gluconate => glycerate−3P
M00307__Pyruvate oxidation, pyruvate => acetyl−CoA
M00011__Citrate cycle, second carbon oxidation, 2−oxoglutarate => oxaloacetate
M00010__Citrate cycle, first carbon oxidation, oxaloacetate => 2−oxoglutarate
M00009__Citrate cycle (TCA cycle, Krebs cycle)
M00008__Entner−Doudoroff pathway, glucose−6P => glyceraldehyde−3P + pyruvate
M00007__Pentose phosphate pathway, non−oxidative phase, fructose 6P => ribose 5P
M00006__Pentose phosphate pathway, oxidative phase, glucose 6P => ribulose 5P
M00005__PRPP biosynthesis, ribose 5P => PRPP
M00004__Pentose phosphate pathway (Pentose phosphate cycle)
M00003__Gluconeogenesis, oxaloacetate => fructose−6P
M00002__Glycolysis, core module involving three−carbon compounds
M00001__Glycolysis (Embden−Meyerhof pathway), glucose => pyruvate
M00854__Glycogen biosynthesis, glucose−1P => glycogen/starch
M00741__Propanoyl−CoA metabolism, propanoyl−CoA => succinyl−CoA
M00632__Galactose degradation, Leloir pathway, galactose => alpha−D−glucose−1P
M00631__D−Galacturonate degradation (bacteria), D−galacturonate => pyruvate + D−glyceraldehyde 3P
M00565__Trehalose biosynthesis, D−glucose 1P => trehalose
M00554__Nucleotide sugar biosynthesis, galactose => UDP−galactose
M00552__D−galactonate degradation, De Ley−Doudoroff pathway, D−galactonate => glycerate−3P
M00550__Ascorbate degradation, ascorbate => D−xylulose−5P
M00549__Nucleotide sugar biosynthesis, glucose => UDP−glucose
M00373__Ethylmalonyl pathway
M00061__D−Glucuronate degradation, D−glucuronate => pyruvate + D−glyceraldehyde 3P
M00013__Malonate semialdehyde pathway, propanoyl−CoA => acetyl−CoA
M00012__Glyoxylate cycle
M00417__Cytochrome o ubiquinol oxidase
M00416__Cytochrome aa3−600 menaquinol oxidase
M00159__V/A−type ATPase, prokaryotes
M00157__F−type ATPase, prokaryotes and chloroplasts
M00156__Cytochrome c oxidase, cbb3−type
M00155__Cytochrome c oxidase, prokaryotes
M00151__Cytochrome bc1 complex respiratory unit
M00150__Fumarate reductase, prokaryotes
M00149__Succinate dehydrogenase, prokaryotes
M00145__NAD(P)H:quinone oxidoreductase, chloroplasts and cyanobacteria
M00144__NADH:quinone oxidoreductase, prokaryotes
M00579__Phosphate acetyltransferase−acetate kinase pathway, acetyl−CoA => acetate
M00173__Reductive citrate cycle (Arnon−Buchanan cycle)
M00172__C4−dicarboxylic acid cycle, NADP − malic enzyme type
M00169__CAM (Crassulacean acid metabolism), light
M00168__CAM (Crassulacean acid metabolism), dark
M00167__Reductive pentose phosphate cycle, glyceraldehyde−3P => ribulose−5P
M00166__Reductive pentose phosphate cycle, ribulose−5P => glyceraldehyde−3P
M00165__Reductive pentose phosphate cycle (Calvin cycle)
M00378__F420 biosynthesis
M00346__Formaldehyde assimilation, serine pathway
M00345__Formaldehyde assimilation, ribulose monophosphate pathway
M00174__Methane oxidation, methanotroph, methane => formaldehyde
M00531__Assimilatory nitrate reduction, nitrate => ammonia
M00530__Dissimilatory nitrate reduction, nitrate => ammonia
M00597__Anoxygenic photosystem II
M00163__Photosystem I
M00161__Photosystem II
M00596__Dissimilatory sulfate reduction, sulfate => H2S
M00595__Thiosulfate oxidation by SOX complex, thiosulfate => sulfate
M00176__Assimilatory sulfate reduction, sulfate => H2S
M00817__Lantibiotic transport system
M00813__Lantibiotic transport system
M00762__Copper−processing system
M00747__Bacitracin transport system
M00634__Oleandomycin transport system
M00584__Acetoin utilization transport system
M00320__Lipopolysaccharide export system
M00315__Uncharacterized ABC transport system
M00314__Bacitracin transport system
M00259__Heme transport system
M00258__Putative ABC transport system
M00256__Cell division transport system
M00255__Lipoprotein−releasing system
M00254__ABC−2 type transport system
M00253__Sodium transport system
M00252__Lipooligosaccharide transport system
M00251__Teichoic acid transport system
M00250__Lipopolysaccharide transport system
M00249__Capsular polysaccharide transport system
M00224__Fluoroquinolone transport system
M00360__Aminoacyl−tRNA biosynthesis, prokaryotes
M00359__Aminoacyl−tRNA biosynthesis, eukaryotes
M00429__Competence−related DNA transformation transporter
M00336__Twin−arginine translocation (Tat) system
M00335__Sec (secretion) system
M00333__Type IV secretion system
M00331__Type II general secretion pathway
M00330__Adhesin protein transport system
M00765__Multidrug resistance, efflux pump Bmr
M00738__Bacitracin resistance, BceAB transporter
M00720__Multidrug resistance, efflux pump VexEF−TolC
M00717__Multidrug resistance, efflux pump NorA
M00715__Lincosamide resistance, efflux pump LmrB
M00712__Multidrug resistance, efflux pump YkkCD
M00710__Multidrug resistance, efflux pump EbrAB
M00707__Multidrug resistance, MdlAB/SmdAB transporter
M00647__Multidrug resistance, efflux pump AcrAB−TolC/SmeDEF
M00754__Nisin resistance, phage shock protein homolog LiaH
M00743__Aminoglycoside resistance, protease HtpX
M00742__Aminoglycoside resistance, protease FtsH
M00728__Cationic antimicrobial peptide (CAMP) resistance, envelope protein folding and degrading factors DegP and DsbA
M00727__Cationic antimicrobial peptide (CAMP) resistance, N−acetylmuramoyl−L−alanine amidase AmiA and AmiC
M00628__beta−Lactam resistance, AmpC system
Biosynthesis of other secondary metabolites
Biosynthesis of other secondary metabolites
Central carbohydrate metabolism
Carbohydrate metabolism
Other carbohydrate metabolism
Carbohydrate metabolism
ATP synthesis
Energy metabolism
Carbon fixation
Energy metabolism
Methane metabolism
Energy metabolism
Nitrogen metabolism
Energy metabolism
Photosynthesis
Energy metabolism
Sulfur metabolism
Energy metabolism
ABC−2 type and other transport systems
Environmental information processing
Aminoacyl tRNA Metabolism
Environmental information processing
Bacterial secretion system
Environmental information processing
Drug efflux transporter/pump
Environmental information processing
Drug resistance
Environmental information processing

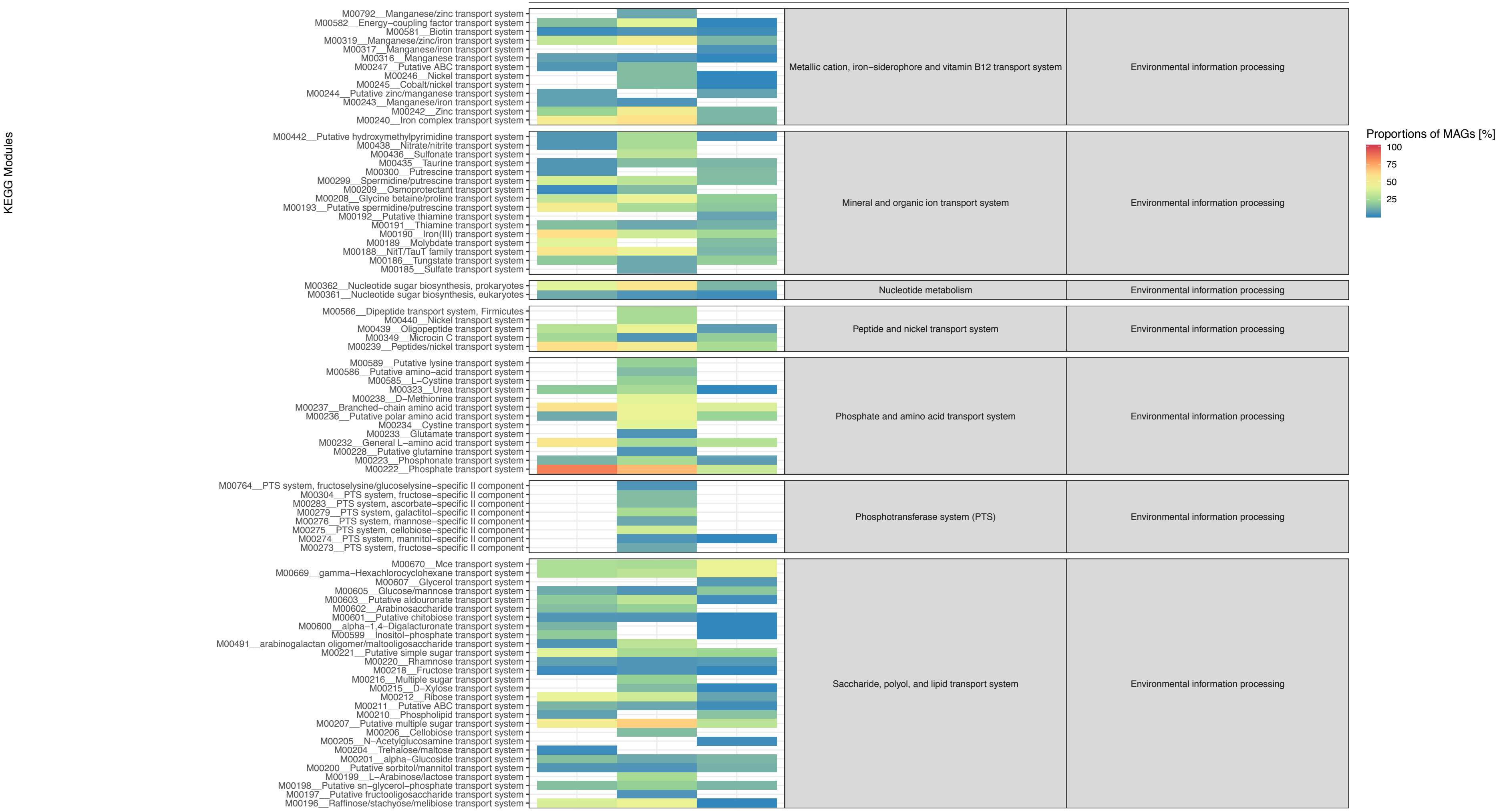
KEGG Modules
M00792__Manganese/zinc transport system
M00582__Energy−coupling factor transport system
M00581__Biotin transport system
M00319__Manganese/zinc/iron transport system
M00317__Manganese/iron transport system
M00316__Manganese transport system
M00247__Putative ABC transport system
M00246__Nickel transport system
M00245__Cobalt/nickel transport system
M00244__Putative zinc/manganese transport system
M00243__Manganese/iron transport system
M00242__Zinc transport system
M00240__Iron complex transport system
Metallic cation, iron−siderophore and vitamin B12 transport system
Environmental information processing
M00442__Putative hydroxymethylpyrimidine transport system
M00438__Nitrate/nitrite transport system
M00436__Sulfonate transport system
M00435__Taurine transport system
M00300__Putrescine transport system
M00299__Spermidine/putrescine transport system
M00209__Osmoprotectant transport system
M00208__Glycine betaine/proline transport system
M00193__Putative spermidine/putrescine transport system
M00192__Putative thiamine transport system
M00191__Thiamine transport system
M00190__Iron(III) transport system
M00189__Molybdate transport system
M00188__NitT/TauT family transport system
M00186__Tungstate transport system
M00185__Sulfate transport system
Mineral and organic ion transport system
Environmental information processing
M00362__Nucleotide sugar biosynthesis, prokaryotes
M00361__Nucleotide sugar biosynthesis, eukaryotes
Nucleotide metabolism
Environmental information processing
M00566__Dipeptide transport system, Firmicutes
M00440__Nickel transport system
M00439__Oligopeptide transport system
M00349__Microcin C transport system
M00239__Peptides/nickel transport system
Peptide and nickel transport system
Environmental information processing
M00589__Putative lysine transport system
M00586__Putative amino−acid transport system
M00585__L−Cystine transport system
M00323__Urea transport system
M00238__D−Methionine transport system
M00237__Branched−chain amino acid transport system
M00236__Putative polar amino acid transport system
M00234__Cystine transport system
M00233__Glutamate transport system
M00232__General L−amino acid transport system
M00228__Putative glutamine transport system
M00223__Phosphonate transport system
M00222__Phosphate transport system
Phosphate and amino acid transport system
Environmental information processing
M00764__PTS system, fructoselysine/glucoselysine−specific II component
M00304__PTS system, fructose−specific II component
M00283__PTS system, ascorbate−specific II component
M00279__PTS system, galactitol−specific II component
M00276__PTS system, mannose−specific II component
M00275__PTS system, cellobiose−specific II component
M00274__PTS system, mannitol−specific II component
M00273__PTS system, fructose−specific II component
Phosphotransferase system (PTS)
Environmental information processing
M00670__Mce transport system
M00669__gamma−Hexachlorocyclohexane transport system
M00607__Glycerol transport system
M00605__Glucose/mannose transport system
M00603__Putative aldouronate transport system
M00602__Arabinosaccharide transport system
M00601__Putative chitobiose transport system
M00600__alpha−1,4−Digalacturonate transport system
M00599__Inositol−phosphate transport system
M00491__arabinogalactan oligomer/maltooligosaccharide transport system
M00221__Putative simple sugar transport system
M00220__Rhamnose transport system
M00218__Fructose transport system
M00216__Multiple sugar transport system
M00215__D−Xylose transport system
M00212__Ribose transport system
M00211__Putative ABC transport system
M00210__Phospholipid transport system
M00207__Putative multiple sugar transport system
M00206__Cellobiose transport system
M00205__N−Acetylglucosamine transport system
M00204__Trehalose/maltose transport system
M00201__alpha−Glucoside transport system
M00200__Putative sorbitol/mannitol transport system
M00199__L−Arabinose/lactose transport system
M00198__Putative sn−glycerol−phosphate transport system
M00197__Putative fructooligosaccharide transport system
M00196__Raffinose/stachyose/melibiose transport system
Saccharide, polyol, and lipid transport system
Environmental information processing
Proportions of MAGs [%]
100
75
50
25

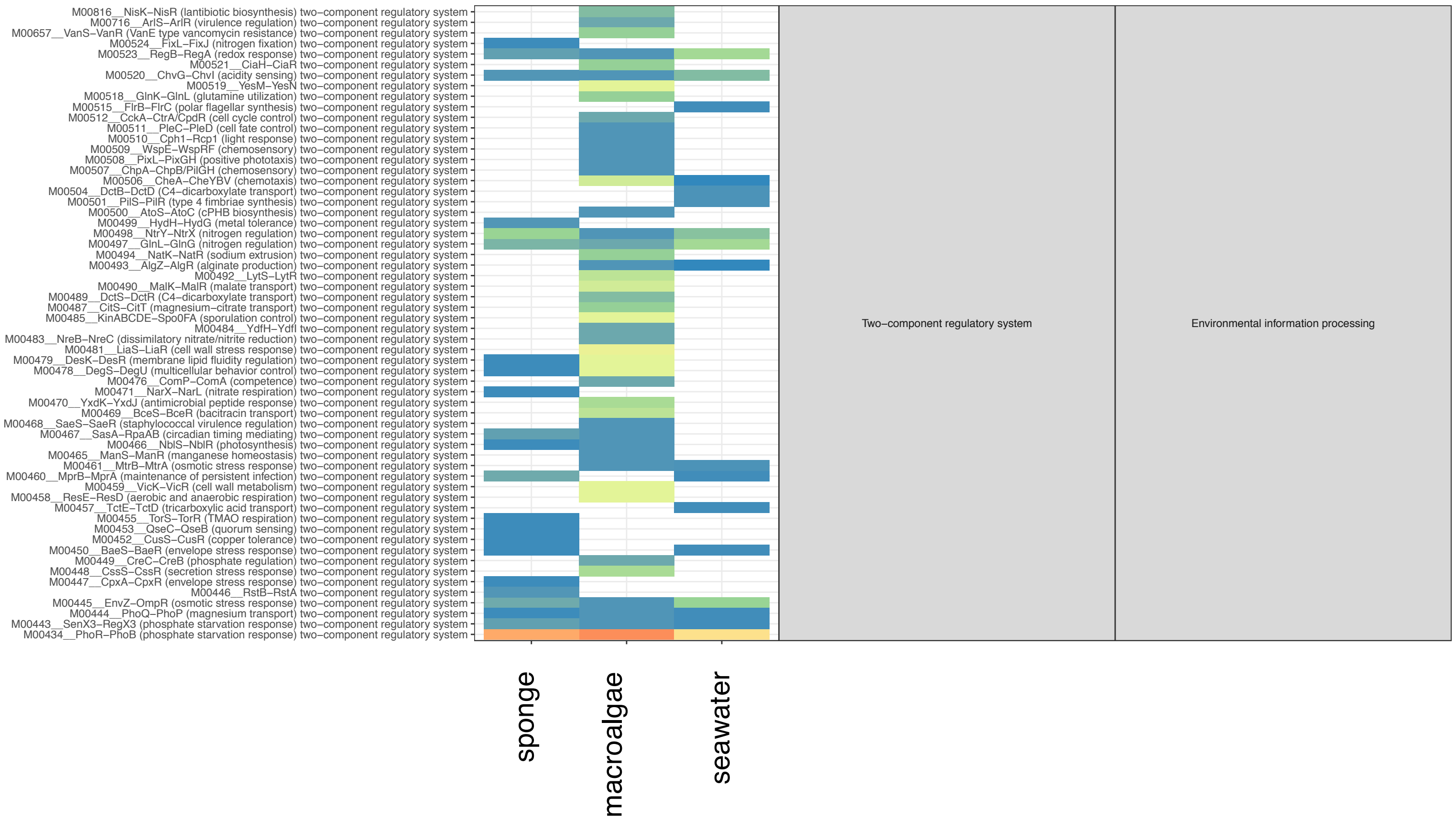

Figure S6. Unique and shared KEGG Modules involved in carbohydrate metabolism, energy metabolism, processing of environmental information and production of other secondary metabolites of sponge, algae and seawater microbiomes. Colour represents the relative proportions of MAGs [in %] in a habitat (sponge, macroalgae and seawater) possessing a certain function.

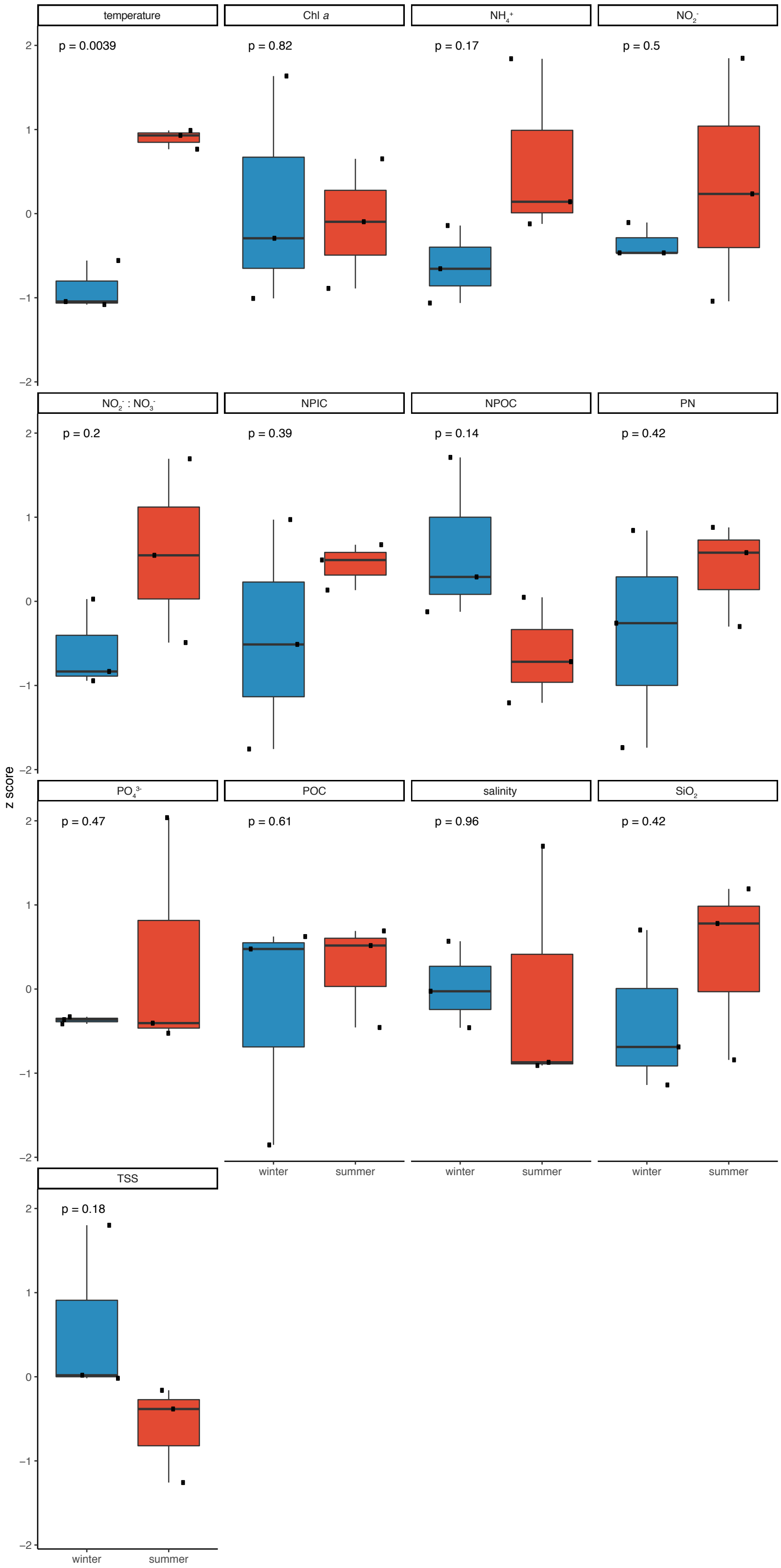

Figure S7. Seasonal variation of environmental parameters. All data were z-score standardised and variation between winter (Jun-16, Aug-16, Oct-16) and summer (Dec-16, Feb-16, Mar-16) was analysed using a t-test.

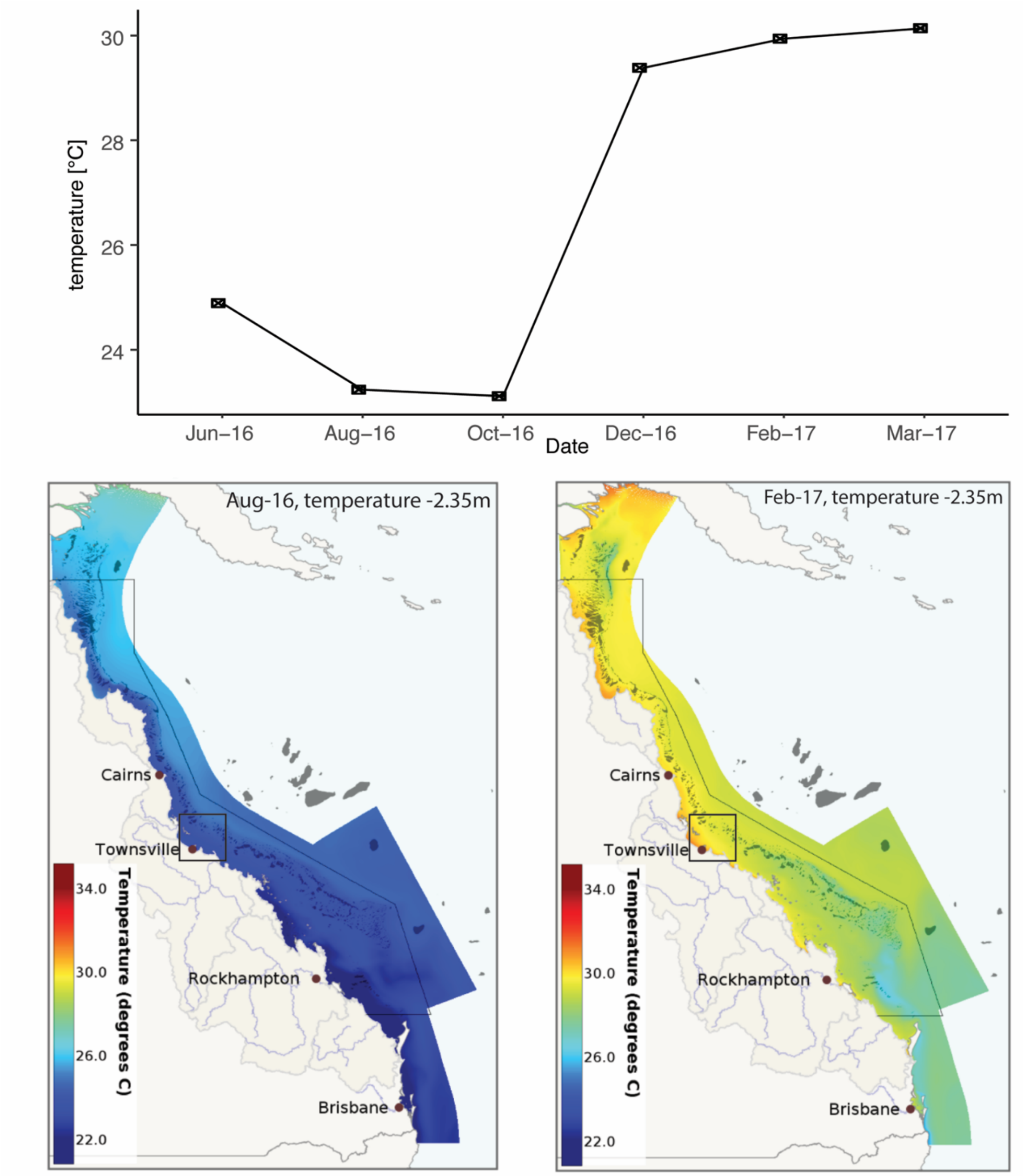

Figure S8. Seawater temperature variation between winter (June, August, October) and summer (December, February, March) at the sampling location (Geoffrey Bay, Magnetic Island, Australia). Monthly average seawater temperature in August 2016 and February 2017 along the Great Barrier Reef. Seawater temperature data were retrieved from the eReefs database (https://aims.ereefs.org.au/).

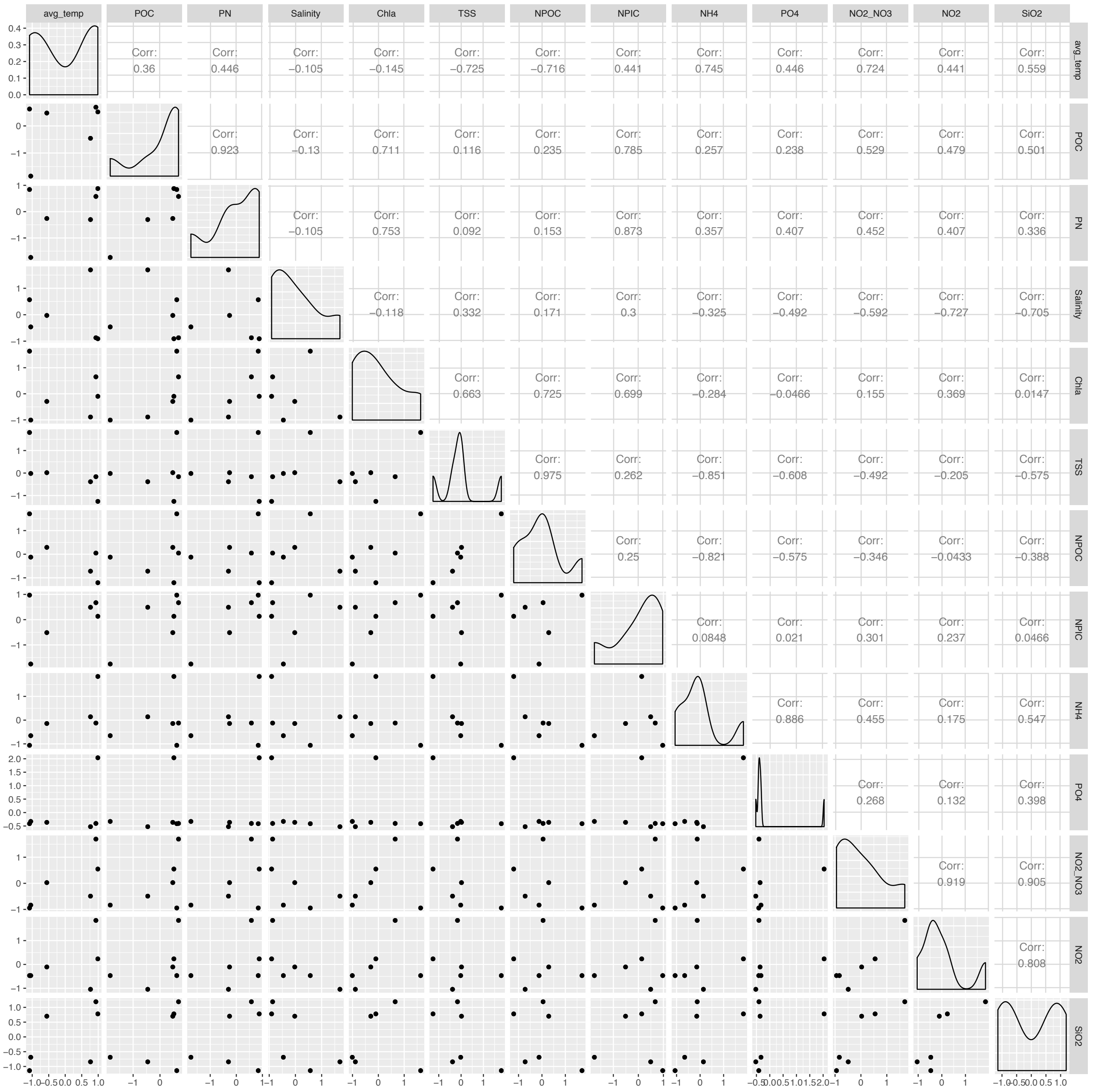

Figure S9. Co-linearity of environmental metadata collected at Geoffrey Bay (Magnetic Island) in (Jun-16, Aug-16, Oct-16, Dec-16, Feb-16, Mar-16 calculated with Pearson correlation (co-linearity threshold: > 0.7 or < -0.7).

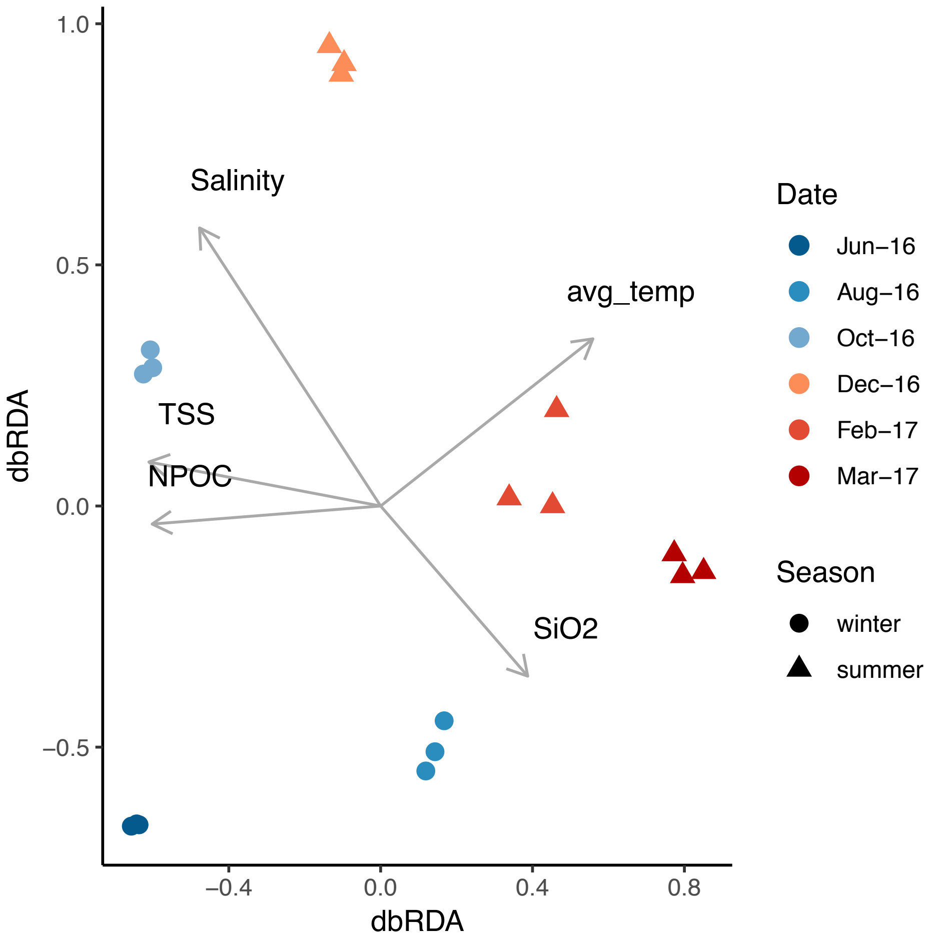

Figure S10. Distance-based redundancy analysis (dbRDA) displaying the influence of significant environmental drivers on the MAGs$_{95\%ANI}$ community profiles for seawater samples (including additional sampling time points of the seawater samples). Temperature (avg_temp), salinity, total suspended solid concentration (TSS), non-purgeable organic carbon (NPOC) concentration, and silica (SiO_2) concentration in the seawater significantly explained 96.6% of the observed MAGs$_{95\%ANI}$ community variation. Co-linear environmental parameters are displayed in Figure S9.

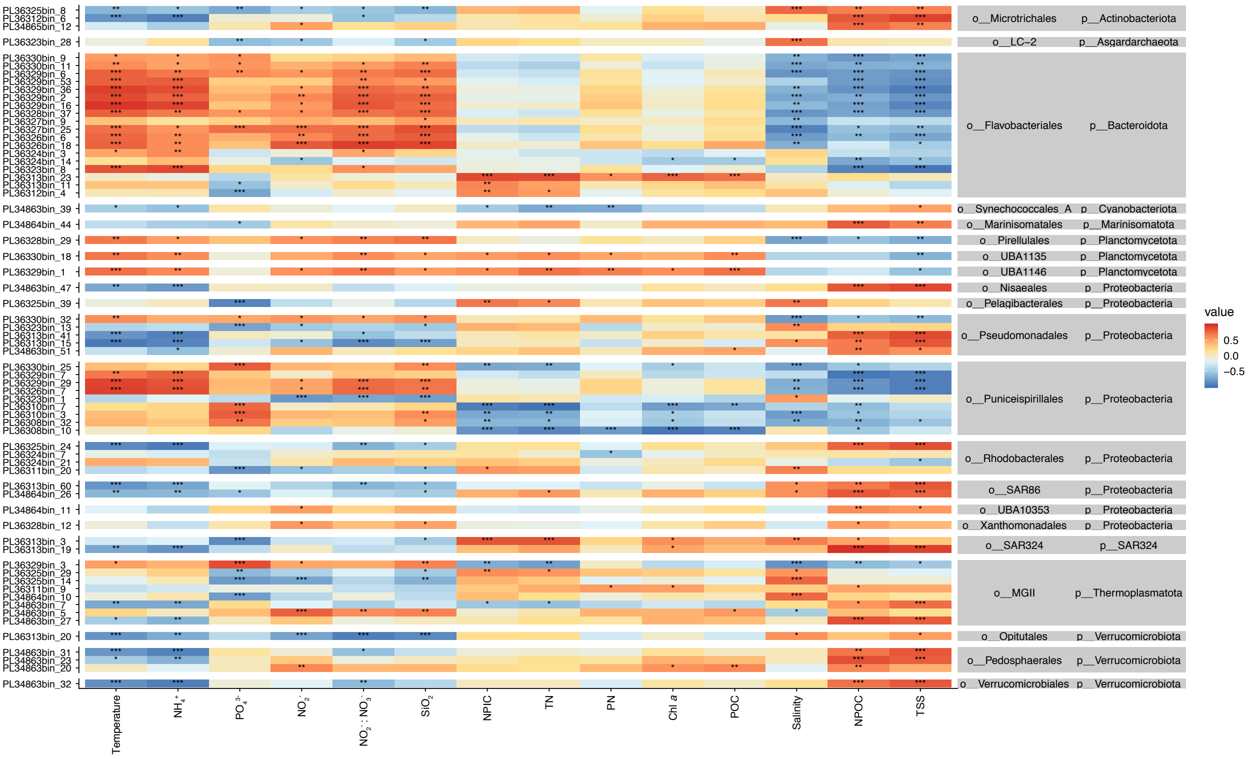

Figure S11. Correlation between the relative abundance of $\text{MAGs}_{95\%\text{ANI}}$ and environmental parameters. Correlations are based on Spearman's rank correlation coefficient (negative correlations are indicated by values < 0, positive correlations are indicated by values > 0). Significant correlations are expressed as * (p > 0.05), ** (p > 0.01), and *** (p > 0.001).

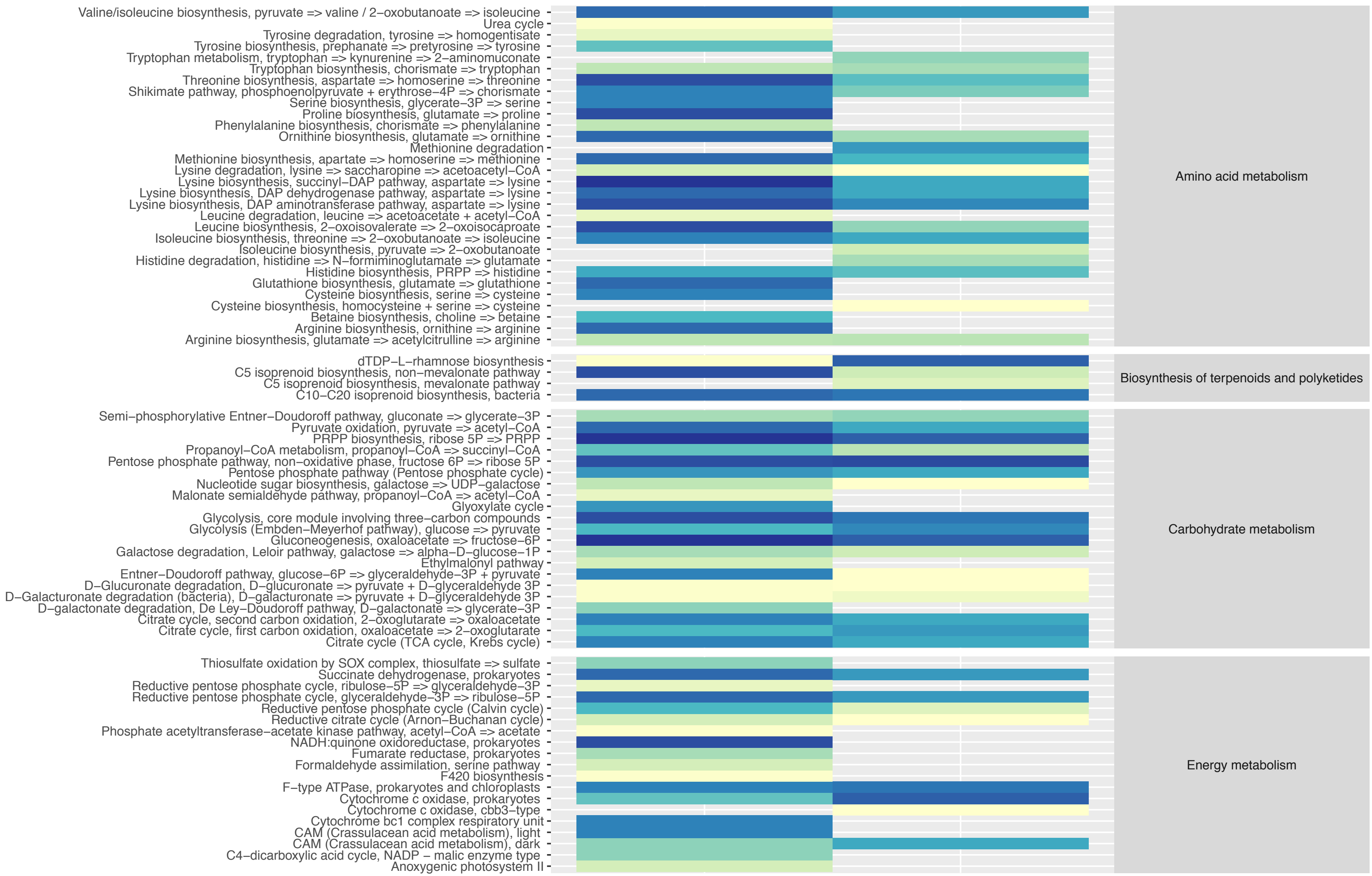
Valine/isoleucine biosynthesis, pyruvate => valine / 2–oxobutanoate => isoleucine
Urea cycle
Tyrosine degradation, tyrosine => homogentisate
Tyrosine biosynthesis, prephanate => pretyrosine => tyrosine
Tryptophan metabolism, tryptophan => kynurenine => 2–aminomuconate
Tryptophan biosynthesis, chorismate => tryptophan
Threonine biosynthesis, aspartate => homoserine => threonine
Shikimate pathway, phosphoenolpyruvate + erythrose–4P => chorismate
Serine biosynthesis, glycerate–3P => serine
Proline biosynthesis, glutamate => proline
Phenylalanine biosynthesis, chorismate => phenylalanine
Ornithine biosynthesis, glutamate => ornithine
Methionine degradation
Methionine biosynthesis, apartate => homoserine => methionine
Lysine degradation, lysine => saccharopine => acetoacetyl–CoA
Lysine biosynthesis, succinyl–DAP pathway, aspartate => lysine
Lysine biosynthesis, DAP dehydrogenase pathway, aspartate => lysine
Lysine biosynthesis, DAP aminotransferase pathway, aspartate => lysine
Leucine degradation, leucine => acetoacetate + acetyl–CoA
Leucine biosynthesis, 2–oxoisovalerate => 2–oxoisocaproate
Isoleucine biosynthesis, threonine => 2–oxobutanoate => isoleucine
Isoleucine biosynthesis, pyruvate => 2–oxobutanoate
Histidine degradation, histidine => N–formiminoglutamate => glutamate
Histidine biosynthesis, PRPP => histidine
Glutathione biosynthesis, glutamate => glutathione
Cysteine biosynthesis, serine => cysteine
Cysteine biosynthesis, homocysteine + serine => cysteine
Betaine biosynthesis, choline => betaine
Arginine biosynthesis, ornithine => arginine
Arginine biosynthesis, glutamate => acetylcitrulline => arginine
Amino acid metabolism
dTDP–L–rhamnose biosynthesis
C5 isoprenoid biosynthesis, non–mevalonate pathway
C5 isoprenoid biosynthesis, mevalonate pathway
C10–C20 isoprenoid biosynthesis, bacteria
Biosynthesis of terpenoids and polyketides
Semi–phosphorylative Entner–Doudoroff pathway, gluconate => glycerate–3P
Pyruvate oxidation, pyruvate => acetyl–CoA
PRPP biosynthesis, ribose 5P => PRPP
Propanoyl–CoA metabolism, propanoyl–CoA => succinyl–CoA
Pentose phosphate pathway, non–oxidative phase, fructose 6P => ribose 5P
Pentose phosphate pathway (Pentose phosphate cycle)
Nucleotide sugar biosynthesis, galactose => UDP–galactose
Malonate semialdehyde pathway, propanoyl–CoA => acetyl–CoA
Glyoxylate cycle
Glycolysis, core module involving three–carbon compounds
Glycolysis (Embden–Meyerhof pathway), glucose => pyruvate
Gluconeogenesis, oxaloacetate => fructose–6P
Galactose degradation, Leloir pathway, galactose => alpha–D–glucose–1P
Ethylmalonyl pathway
Entner–Doudoroff pathway, glucose–6P => glyceraldehyde–3P + pyruvate
D–Glucuronate degradation, D–glucuronate => pyruvate + D–glyceraldehyde 3P
D–Galacturonate degradation (bacteria), D–galacturonate => pyruvate + D–glyceraldehyde 3P
D–galactonate degradation, De Ley–Doudoroff pathway, D–galactonate => glycerate–3P
Citrate cycle, second carbon oxidation, 2–oxoglutarate => oxaloacetate
Citrate cycle, first carbon oxidation, oxaloacetate => 2–oxoglutarate
Citrate cycle (TCA cycle, Krebs cycle)
Carbohydrate metabolism
Thiosulfate oxidation by SOX complex, thiosulfate => sulfate
Succinate dehydrogenase, prokaryotes
Reductive pentose phosphate cycle, ribulose–5P => glyceraldehyde–3P
Reductive pentose phosphate cycle, glyceraldehyde–3P => ribulose–5P
Reductive pentose phosphate cycle (Calvin cycle)
Reductive citrate cycle (Arnon–Buchanan cycle)
Phosphate acetyltransferase–acetate kinase pathway, acetyl–CoA => acetate
NADH:quinone oxidoreductase, prokaryotes
Fumarate reductase, prokaryotes
Formaldehyde assimilation, serine pathway
F420 biosynthesis
F–type ATPase, prokaryotes and chloroplasts
Cytochrome c oxidase, prokaryotes
Cytochrome c oxidase, cbb3–type
Cytochrome bc1 complex respiratory unit
CAM (Crassulacean acid metabolism), light
CAM (Crassulacean acid metabolism), dark
C4–dicarboxylic acid cycle, NADP – malic enzyme type
Anoxygenic photosystem II
Energy metabolism

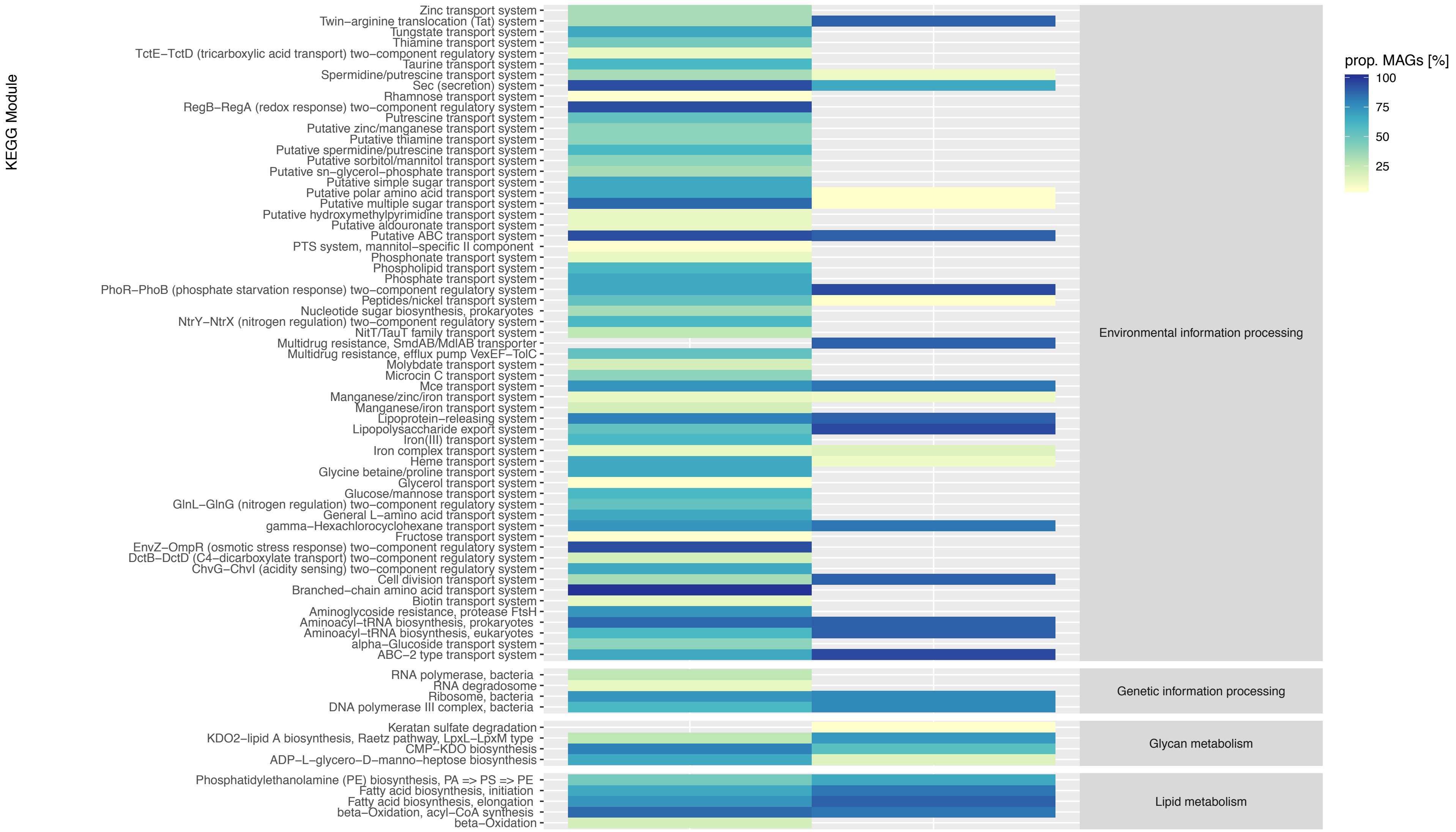

KEGG Module
Zinc transport system
Twin−arginine translocation (Tat) system
Tungstate transport system
Thiamine transport system
TctE−TctD (tricarboxylic acid transport) two−component regulatory system
Taurine transport system
Spermidine/putrescine transport system
Sec (secretion) system
Rhamnose transport system
RegB−RegA (redox response) two−component regulatory system
Putrescine transport system
Putative zinc/manganese transport system
Putative thiamine transport system
Putative spermidine/putrescine transport system
Putative sorbitol/mannitol transport system
Putative sn−glycerol−phosphate transport system
Putative simple sugar transport system
Putative polar amino acid transport system
Putative multiple sugar transport system
Putative hydroxymethylpyrimidine transport system
Putative aldouronate transport system
Putative ABC transport system
PTS system, mannitol−specific II component
Phosphonate transport system
Phospholipid transport system
Phosphate transport system
PhoR−PhoB (phosphate starvation response) two−component regulatory system
Peptides/nickel transport system
Nucleotide sugar biosynthesis, prokaryotes
NtrY−NtrX (nitrogen regulation) two−component regulatory system
NitT/TauT family transport system
Multidrug resistance, SmdAB/MdlAB transporter
Multidrug resistance, efflux pump VexEF−TolC
Molybdate transport system
Microcin C transport system
Mce transport system
Manganese/zinc/iron transport system
Manganese/iron transport system
Lipoprotein−releasing system
Lipopolysaccharide export system
Iron(III) transport system
Iron complex transport system
Heme transport system
Glycine betaine/proline transport system
Glycerol transport system
Glucose/mannose transport system
GlnL−GlnG (nitrogen regulation) two−component regulatory system
General L−amino acid transport system
gamma−Hexachlorocyclohexane transport system
Fructose transport system
EnvZ−OmpR (osmotic stress response) two−component regulatory system
DctB−DctD (C4−dicarboxylate transport) two−component regulatory system
ChvG−ChvI (acidity sensing) two−component regulatory system
Cell division transport system
Branched−chain amino acid transport system
Biotin transport system
Aminoglycoside resistance, protease FtsH
Aminoacyl−tRNA biosynthesis, prokaryotes
Aminoacyl−tRNA biosynthesis, eukaryotes
alpha−Glucoside transport system
ABC−2 type transport system
Environmental information processing
RNA polymerase, bacteria
RNA degradosome
Ribosome, bacteria
DNA polymerase III complex, bacteria
Genetic information processing
Keratan sulfate degradation
KDO2−lipid A biosynthesis, Raetz pathway, LpxL−LpxM type
CMP−KDO biosynthesis
ADP−L−glycero−D−manno−heptose biosynthesis
Glycan metabolism
Phosphatidylethanolamine (PE) biosynthesis, PA => PS => PE
Fatty acid biosynthesis, initiation
Fatty acid biosynthesis, elongation
beta−Oxidation, acyl−CoA synthesis
beta−Oxidation
Lipid metabolism
prop. MAGs [%]
100
75
50
25

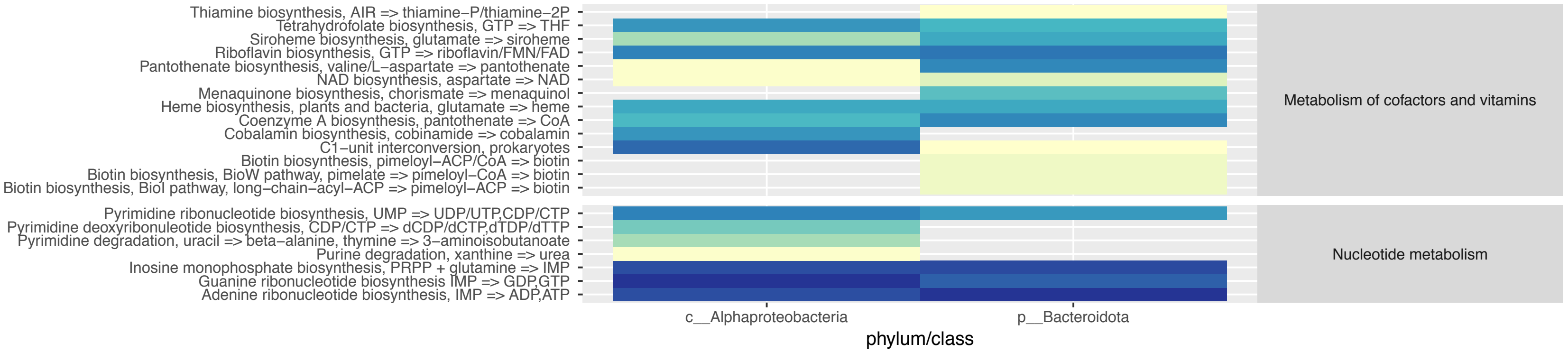

Figure S12. KEGG Modules of Alphaproteobacteria and Bacteroidota MAGs$_{95\%ANI}$ derived from seawater. Colour indicates the number of MAGs$_{95\%ANI}$ (in %) that were associated with individual KEGG Modules.

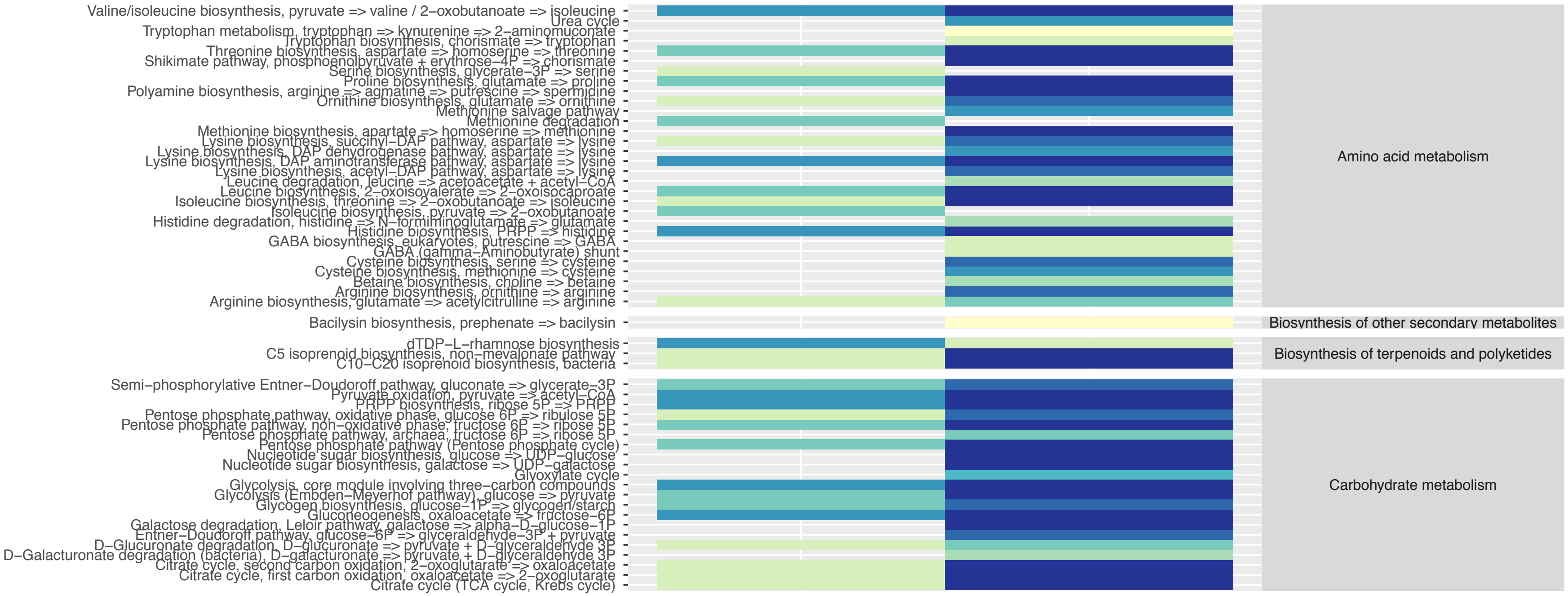

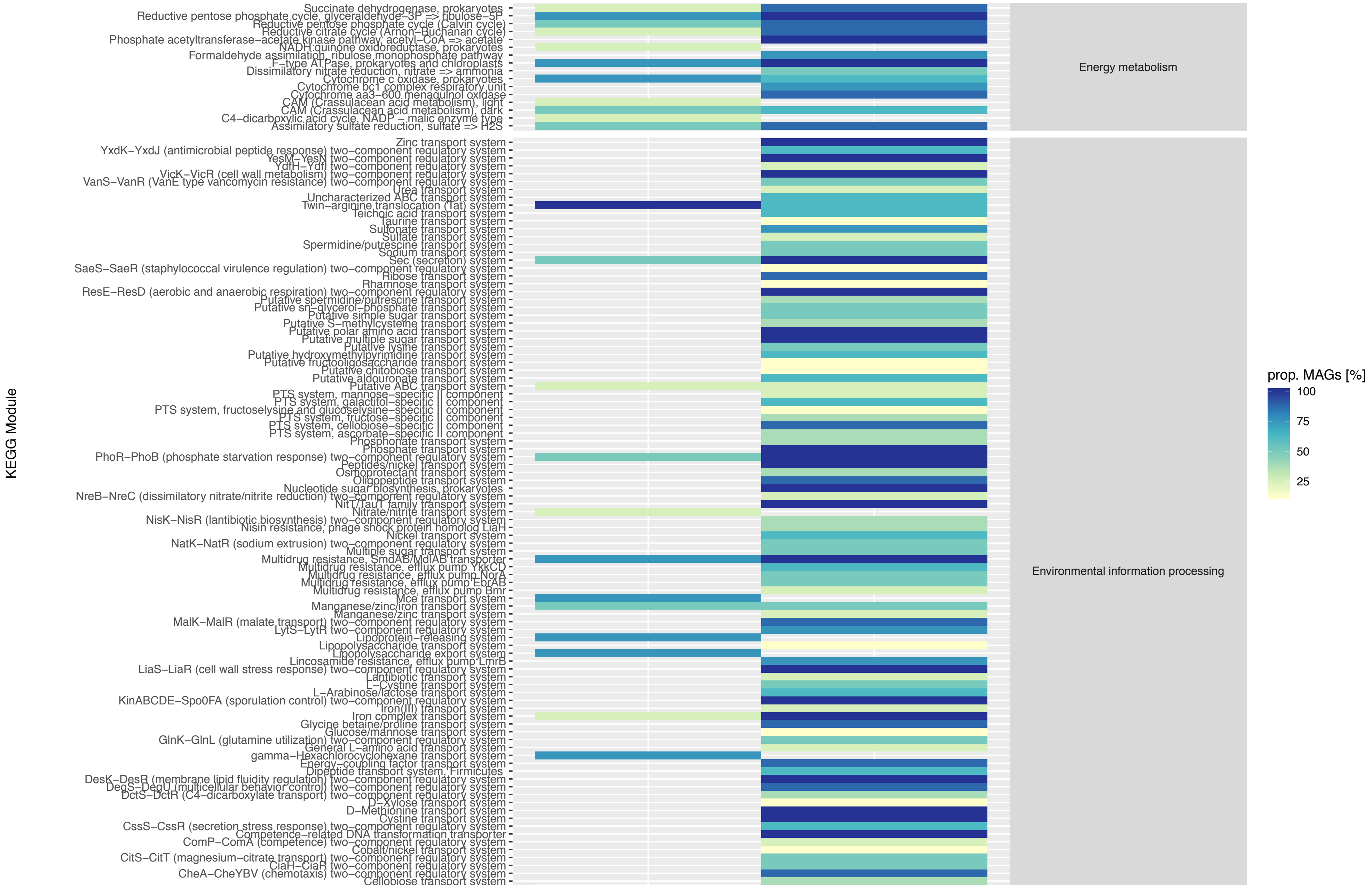

Succinate dehydrogenase, prokaryotes
Reductive pentose phosphate cycle, glyceraldehyde−3P => ribulose−5P
Reductive pentose phosphate cycle (Calvin cycle)
Reductive citrate cycle (Arnon−Buchanan cycle)
Phosphate acetyltransferase−acetate kinase pathway, acetyl−CoA => acetate
NADH:quinone oxidoreductase, prokaryotes
Formaldehyde assimilation, ribulose monophosphate pathway
F−type ATPase, prokaryotes and chloroplasts
Dissimilatory nitrate reduction, nitrate => ammonia
Cytochrome c oxidase, prokaryotes
Cytochrome bc1 complex respiratory unit
Cytochrome aa3−600 menaquinol oxidase
CAM (Crassulacean acid metabolism), light
CAM (Crassulacean acid metabolism), dark
C4−dicarboxylic acid cycle, NADP − malic enzyme type
Assimilatory sulfate reduction, sulfate => H2S
Zinc transport system
YxdK−YxdJ (antimicrobial peptide response) two−component regulatory system
YesM−YesN two−component regulatory system
YdfH−YdfI two−component regulatory system
VicK−VicR (cell wall metabolism) two−component regulatory system
VanS−VanR (VanE type vancomycin resistance) two−component regulatory system
Urea transport system
Uncharacterized ABC transport system
Twin−arginine translocation (Tat) system
Teichoic acid transport system
Taurine transport system
Sulfonate transport system
Sulfate transport system
Spermidine/putrescine transport system
Sodium transport system
Sec (secretion) system
SaeS−SaeR (staphylococcal virulence regulation) two−component regulatory system
Ribose transport system
Rhamnose transport system
ResE−ResD (aerobic and anaerobic respiration) two−component regulatory system
Putative spermidine/putrescine transport system
Putative sn−glycerol−phosphate transport system
Putative simple sugar transport system
Putative S−methylcysteine transport system
Putative polar amino acid transport system
Putative multiple sugar transport system
Putative lysine transport system
Putative hydroxymethylpyrimidine transport system
Putative fructooligosaccharide transport system
Putative chitobiose transport system
Putative aldouronate transport system
Putative ABC transport system
PTS system, mannose−specific II component
PTS system, galactitol−specific II component
PTS system, fructoselysine and glucoselysine−specific II component
PTS system, fructose−specific II component
PTS system, cellobiose−specific II component
PTS system, ascorbate−specific II component
Phosphonate transport system
Phosphate transport system
PhoR−PhoB (phosphate starvation response) two−component regulatory system
Peptides/nickel transport system
Osmoprotectant transport system
Oligopeptide transport system
Nucleotide sugar biosynthesis, prokaryotes
NreB−NreC (dissimilatory nitrate/nitrite reduction) two−component regulatory system
NitT/TauT family transport system
Nitrate/nitrite transport system
NisK−NisR (lantibiotic biosynthesis) two−component regulatory system
Nisin resistance, phage shock protein homolog LiaH
Nickel transport system
NatK−NatR (sodium extrusion) two−component regulatory system
Multiple sugar transport system
Multidrug resistance, SmdAB/MdlAB transporter
Multidrug resistance, efflux pump YkkCD
Multidrug resistance, efflux pump NorA
Multidrug resistance, efflux pump EbrAB
Multidrug resistance, efflux pump Bmr
Mce transport system
Manganese/zinc/iron transport system
Manganese/zinc transport system
MalK−MalR (malate transport) two−component regulatory system
LytS−LytR two−component regulatory system
Lipoprotein−releasing system
Lipopolysaccharide transport system
Lipopolysaccharide export system
Lincosamide resistance, efflux pump LmrB
LiaS−LiaR (cell wall stress response) two−component regulatory system
Lantibiotic transport system
L−Cystine transport system
L−Arabinose/lactose transport system
KinABCDE−Spo0FA (sporulation control) two−component regulatory system
Iron(III) transport system
Iron complex transport system
Glycine betaine/proline transport system
Glucose/mannose transport system
GlnK−GlnL (glutamine utilization) two−component regulatory system
General L−amino acid transport system
gamma−Hexachlorocyclohexane transport system
Energy−coupling factor transport system
Dipeptide transport system, Firmicutes
DesK−DesR (membrane lipid fluidity regulation) two−component regulatory system
DegS−DegU (multicellular behavior control) two−component regulatory system
DctS−DctR (C4−dicarboxylate transport) two−component regulatory system
D−Xylose transport system
D−Methionine transport system
Cystine transport system
CssS−CssR (secretion stress response) two−component regulatory system
Competence−related DNA transformation transporter
ComP−ComA (competence) two−component regulatory system
Cobalt/nickel transport system
CitS−CitT (magnesium−citrate transport) two−component regulatory system
CiaH−CiaR two−component regulatory system
CheA−CheYBV (chemotaxis) two−component regulatory system
Cellobiose transport system
KEGG Module
Energy metabolism
Environmental information processing
prop. MAGs [%]
100
75
50
25

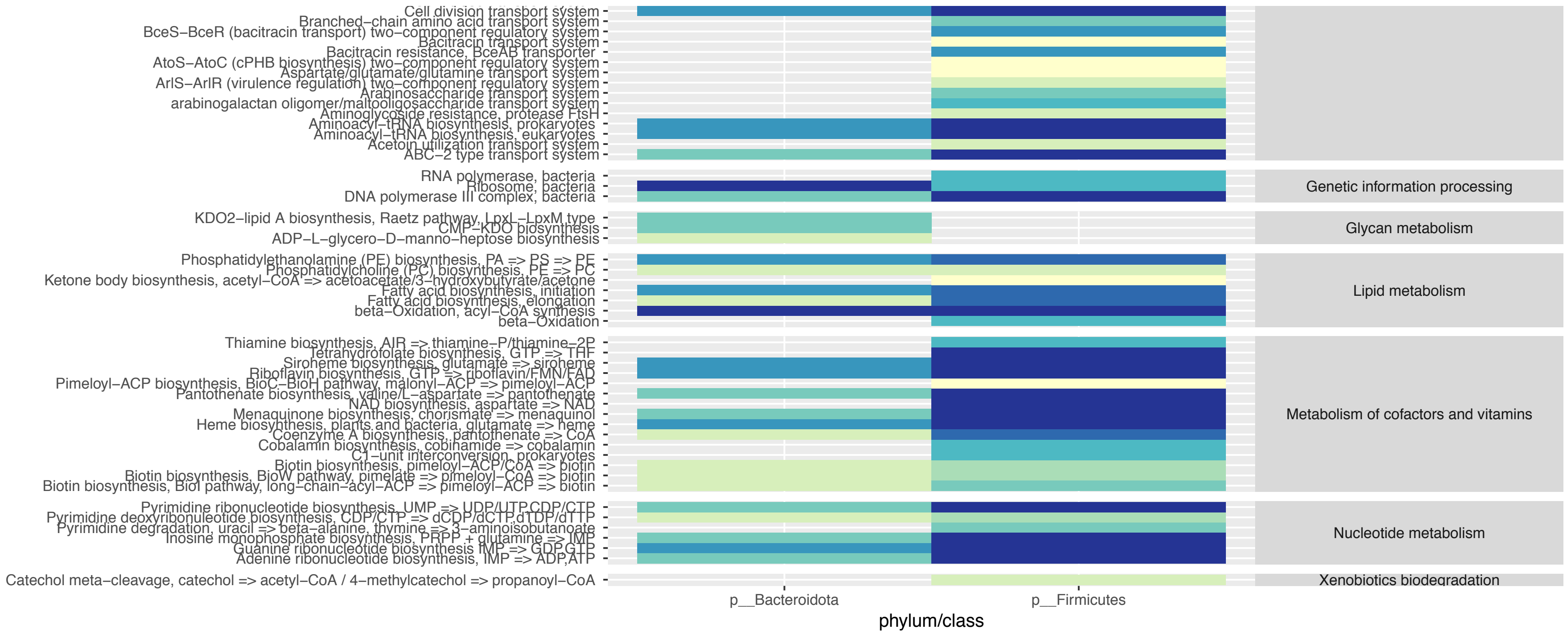

Figure S13. KEGG Modules of Bacteroidota and Firmicutes $MAGs_{95\%ANI}$ derived from macroalgae. Colour indicates the number of $MAGs_{95\%ANI}$ (in %) that were associated with individual KEGG Modules.

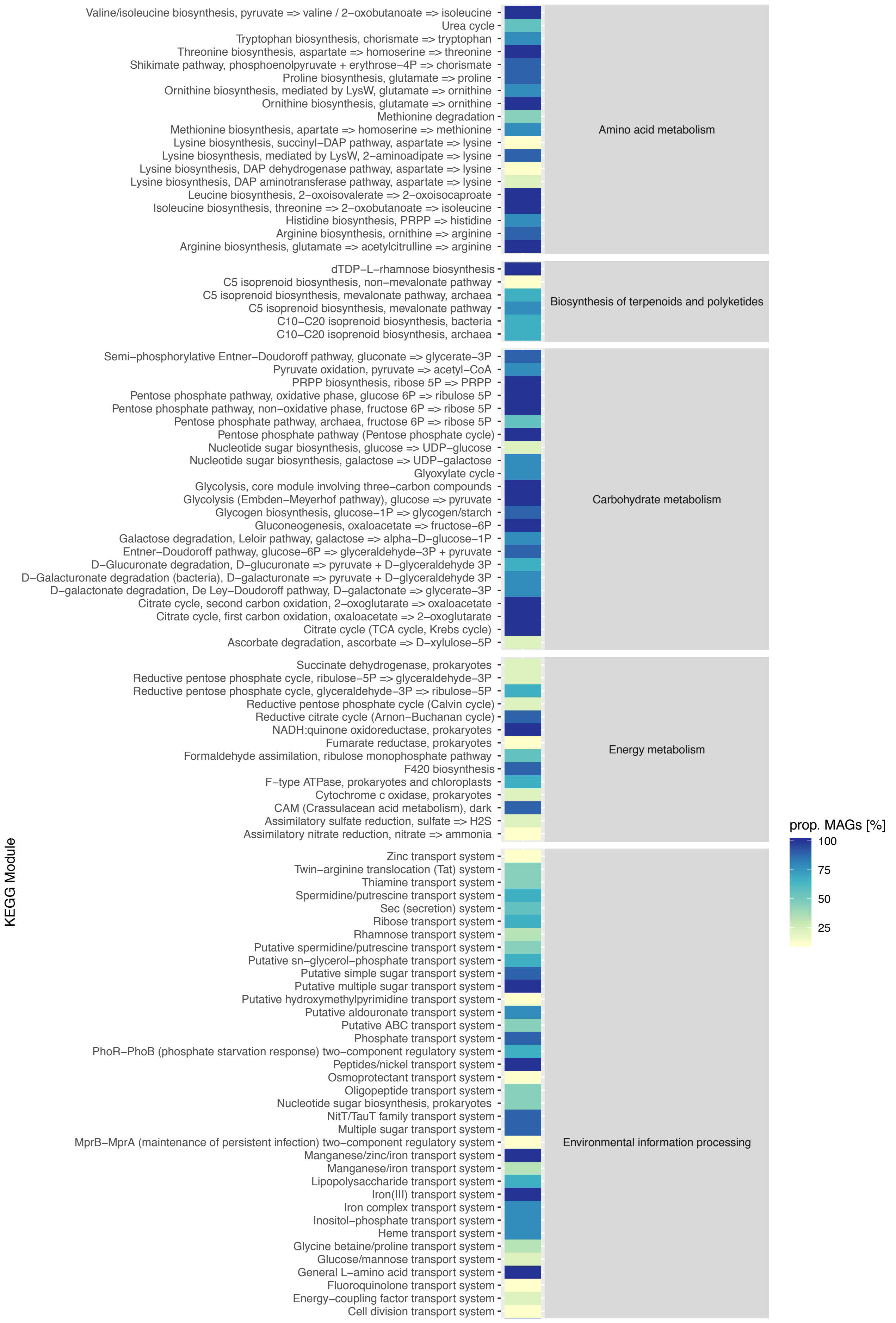

Valine/isoleucine biosynthesis, pyruvate => valine / 2−oxobutanoate => isoleucine
Urea cycle
Tryptophan biosynthesis, chorismate => tryptophan
Threonine biosynthesis, aspartate => homoserine => threonine
Shikimate pathway, phosphoenolpyruvate + erythrose−4P => chorismate
Proline biosynthesis, glutamate => proline
Ornithine biosynthesis, mediated by LysW, glutamate => ornithine
Ornithine biosynthesis, glutamate => ornithine
Methionine degradation
Methionine biosynthesis, apartate => homoserine => methionine
Lysine biosynthesis, succinyl−DAP pathway, aspartate => lysine
Lysine biosynthesis, mediated by LysW, 2−aminoadipate => lysine
Lysine biosynthesis, DAP dehydrogenase pathway, aspartate => lysine
Lysine biosynthesis, DAP aminotransferase pathway, aspartate => lysine
Leucine biosynthesis, 2−oxoisovalerate => 2−oxoisocaproate
Isoleucine biosynthesis, threonine => 2−oxobutanoate => isoleucine
Histidine biosynthesis, PRPP => histidine
Arginine biosynthesis, ornithine => arginine
Arginine biosynthesis, glutamate => acetylcitrulline => arginine
Amino acid metabolism
dTDP−L−rhamnose biosynthesis
C5 isoprenoid biosynthesis, non−mevalonate pathway
C5 isoprenoid biosynthesis, mevalonate pathway, archaea
C5 isoprenoid biosynthesis, mevalonate pathway
C10−C20 isoprenoid biosynthesis, bacteria
C10−C20 isoprenoid biosynthesis, archaea
Biosynthesis of terpenoids and polyketides
Semi−phosphorylative Entner−Doudoroff pathway, gluconate => glycerate−3P
Pyruvate oxidation, pyruvate => acetyl−CoA
PRPP biosynthesis, ribose 5P => PRPP
Pentose phosphate pathway, oxidative phase, glucose 6P => ribulose 5P
Pentose phosphate pathway, non−oxidative phase, fructose 6P => ribose 5P
Pentose phosphate pathway, archaea, fructose 6P => ribose 5P
Pentose phosphate pathway (Pentose phosphate cycle)
Nucleotide sugar biosynthesis, glucose => UDP−glucose
Nucleotide sugar biosynthesis, galactose => UDP−galactose
Glyoxylate cycle
Glycolysis, core module involving three−carbon compounds
Glycolysis (Embden−Meyerhof pathway), glucose => pyruvate
Glycogen biosynthesis, glucose−1P => glycogen/starch
Gluconeogenesis, oxaloacetate => fructose−6P
Galactose degradation, Leloir pathway, galactose => alpha−D−glucose−1P
Entner−Doudoroff pathway, glucose−6P => glyceraldehyde−3P + pyruvate
D−Glucuronate degradation, D−glucuronate => pyruvate + D−glyceraldehyde 3P
D−Galacturonate degradation (bacteria), D−galacturonate => pyruvate + D−glyceraldehyde 3P
D−galactonate degradation, De Ley−Doudoroff pathway, D−galactonate => glycerate−3P
Citrate cycle, second carbon oxidation, 2−oxoglutarate => oxaloacetate
Citrate cycle, first carbon oxidation, oxaloacetate => 2−oxoglutarate
Citrate cycle (TCA cycle, Krebs cycle)
Ascorbate degradation, ascorbate => D−xylulose−5P
Carbohydrate metabolism
Succinate dehydrogenase, prokaryotes
Reductive pentose phosphate cycle, ribulose−5P => glyceraldehyde−3P
Reductive pentose phosphate cycle, glyceraldehyde−3P => ribulose−5P
Reductive pentose phosphate cycle (Calvin cycle)
Reductive citrate cycle (Arnon−Buchanan cycle)
NADH:quinone oxidoreductase, prokaryotes
Fumarate reductase, prokaryotes
Formaldehyde assimilation, ribulose monophosphate pathway
F420 biosynthesis
F−type ATPase, prokaryotes and chloroplasts
Cytochrome c oxidase, prokaryotes
CAM (Crassulacean acid metabolism), dark
Assimilatory sulfate reduction, sulfate => H2S
Assimilatory nitrate reduction, nitrate => ammonia
Energy metabolism
Zinc transport system
Twin−arginine translocation (Tat) system
Thiamine transport system
Spermidine/putrescine transport system
Sec (secretion) system
Ribose transport system
Rhamnose transport system
Putative spermidine/putrescine transport system
Putative sn−glycerol−phosphate transport system
Putative simple sugar transport system
Putative multiple sugar transport system
Putative hydroxymethylpyrimidine transport system
Putative aldouronate transport system
Putative ABC transport system
Phosphate transport system
PhoR−PhoB (phosphate starvation response) two−component regulatory system
Peptides/nickel transport system
Osmoprotectant transport system
Oligopeptide transport system
Nucleotide sugar biosynthesis, prokaryotes
NitT/TauT family transport system
Multiple sugar transport system
MprB−MprA (maintenance of persistent infection) two−component regulatory system
Manganese/zinc/iron transport system
Manganese/iron transport system
Lipopolysaccharide transport system
Iron(III) transport system
Iron complex transport system
Inositol−phosphate transport system
Heme transport system
Glycine betaine/proline transport system
Glucose/mannose transport system
General L−amino acid transport system
Fluoroquinolone transport system
Energy−coupling factor transport system
Cell division transport system
Environmental information processing
KEGG Module
prop. MAGs [%]
100
75
50
25

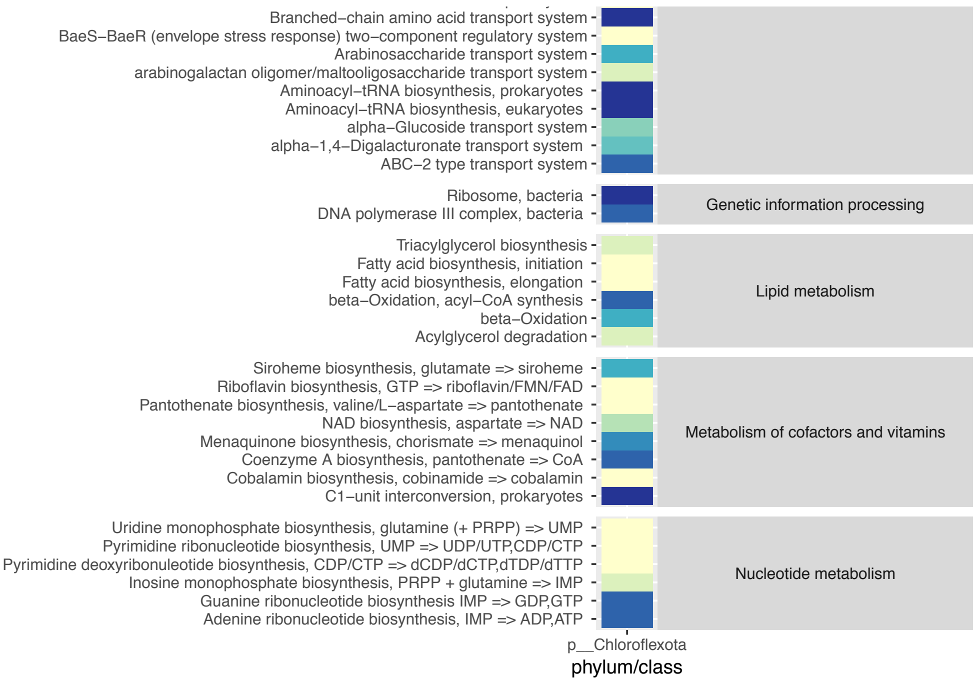

Figure S14. KEGG Modules of Chloroflexota MAGs$_{95\%ANI}$ derived from the sponge *Coscinoderma matthewsi*. Colour indicates the number of MAGs$_{95\%ANI}$ (in %) that were associated with individual KEGG Modules.

Table S1. Sample identifiers for the raw metagenome data at the Australian Microbiome data portal Initiative under the Australian Microbiome project. Raw sequencing data can be downloaded from https://data.bioplatforms.com/organization/about/australian-microbiome.

Host/Habitat	Date sampled	Sample ID - microbial samples		
seawater	2016-06-08	34863	34864	34865
seawater	2016-08-01	36308	36309	36310
seawater	2016-10-10	36311	36312	36313
seawater	2016-12-21	36323	36324	36325
seawater	2017-02-20	36326	36327	36328
seawater	2017-03-18	36329	36330	36331
Coscinoderma matthewsi	2016-08-01	36386	36387	36388
Coscinoderma matthewsi	2017-02-20	36404	36405	36406
Sargassum spp.	2016-08-01	36425	36426	36427
Sargassum spp.	2017-02-20	36439	36440	36441

Table S2. Binning results prior to de-replication

habitat	nr of samples	bins total	bins with quality ≥ 50	Completeness (in %)	Contamination (in %)	Total recovery (in %)
**sponge**	6	271	151	84.01 (± 11.81)	1.97 (± 1.98)	63.5
**macroalgae**	6	215	52	79.65 (± 14.58)	2.04 (± 2.19)	27.7
**seawater**	18	1908	765	76.47 (± 12.35)	1.47 (± 1.44)	35.2

Table S3. Taxonomic affiliations of de-replicated MAGs (based on 95% Average Nucleotide Identity) using GTDB taxonomy.

a) Sponge MAGs$_{95\%ANI}$

Bin_ID	domain	phylum	class	order	family	genus	species
CO36406bin_13	d__Archaea	p__Crenarchaeota	c__Nitrososphaeria	o__Nitrososphaerales	f__Nitrosopumilaceae	g__	
CO36404bin_3	d__Bacteria	p__Chloroflexota	c__Anaerolineae	o__Caldilineales	f__Caldilineaceae	g__bin5	
CO36405bin_14	d__Bacteria	p__Chloroflexota	c__Anaerolineae	o__Caldilineales	f__Caldilineaceae	g__bin5	
CO36388bin_16	d__Bacteria	p__Chloroflexota	c__Anaerolineae	o__SBR1031	f__A4b	g__UBA6055	
CO36386bin_31	d__Bacteria	p__Chloroflexota	c__Anaerolineae	o__SBR1031	f__A4b	g__UBA6055	
CO36386bin_32	d__Bacteria	p__Chloroflexota	c__Anaerolineae	o__SBR1031	f__A4b	g__UBA6055	
CO36388bin_17	d__Bacteria	p__Chloroflexota	c__Anaerolineae	o__SBR1031	f__A4b	g__UBA6055	
CO36386bin_4	d__Bacteria	p__Chloroflexota	c__Anaerolineae	o__SBR1031	f__A4b	g__UBA6055	
CO36387bin_22	d__Bacteria	p__Chloroflexota	c__Anaerolineae	o__SBR1031	f__	g__	
CO36386bin_13	d__Bacteria	p__Chloroflexota	c__UBA2235	o__UBA11872	f__UBA11872	g__	
CO36404bin_8	d__Bacteria	p__Actinobacteriota	c__Acidimicrobiia	o__bin76	f__	g__	
CO36387bin_10	d__Bacteria	p__Actinobacteriota	c__Acidimicrobiia	o__bin76	f__	g__	
CO36386bin_14	d__Bacteria	p__Actinobacteriota	c__Acidimicrobiia	o__Microtrichales	f__TK06	g__	
CO36406bin_26	d__Bacteria	p__Cyanobacteriota	c__Cyanobacteriia	o__Synechococcales_A	f__Cyanobiaceae	g__Synechococcus_C	
CO36386bin_37	d__Bacteria	p__Cyanobacteriota	c__Cyanobacteriia	o__Synechococcales_A	f__Cyanobiaceae	g__Synechococcus_C	
CO36386bin_29	d__Bacteria	p__Cyanobacteriota	c__Cyanobacteriia	o__Synechococcales_A	f__Cyanobiaceae	g__Synechococcus_C	
CO36386bin_10	d__Bacteria	p__Planctomycetota	c__Planctomycetes	o__Pirellulales	f__Pirellulaceae	g__Mariniblastus	
CO36387bin_4	d__Bacteria	p__Planctomycetota	c__Planctomycetes	o__Pirellulales	f__Pirellulaceae	g__Rubripirellula	
CO36405bin_2	d__Bacteria	p__Planctomycetota	c__Planctomycetes	o__Pirellulales	f__UBA1268	g__UBA1268	
CO36405bin_5	d__Bacteria	p__Planctomycetota	c__Planctomycetes	o__Pirellulales	f__UBA1268	g__	
CO36404bin_19	d__Bacteria	p__Acidobacteriota	c__Luteitaleia	o__Luteitaleales	f__UBA8438	g__	
CO36386bin_12	d__Bacteria	p__Acidobacteriota	c__Luteitaleia	o__Luteitaleales	f__UBA8438	g__	
CO36388bin_9	d__Bacteria	p__Acidobacteriota	c__Thermoanaerobaculia	o__	f__	g__	
CO36388bin_1	d__Bacteria	p__Acidobacteriota	c__Thermoanaerobaculia	o__	f__	g__	
CO36406bin_18	d__Bacteria	p__UBP10	c__GR-WP33-30	o__bin18	f__bin18	g__bin18	
CO36406bin_19	d__Bacteria	p__UBP10	c__GR-WP33-30	o__bin18	f__bin18	g__	
CO36405bin_1	d__Bacteria	p__Nitrospirota	c__Nitrospiria	o__Nitrospirales	f__UBA8639	g__bin75	
CO36386bin_35	d__Bacteria	p__Nitrospinota	c__UBA8248	o__UBA8248	f__UBA8248	g__bin107	
CO36404bin_6	d__Bacteria	p__Proteobacteria	c__Alphaproteobacteria	o__UBA828	f__	g__	
CO36404bin_4	d__Bacteria	p__Proteobacteria	c__Alphaproteobacteria	o__Rhodobacterales	f__Rhodobacteraceae	g__bin36	
CO36386bin_9	d__Bacteria	p__Proteobacteria	c__Alphaproteobacteria	o__bin65	f__bin65	g__bin65	
CO36386bin_3	d__Bacteria	p__Proteobacteria	c__Gammaproteobacteria	o__UBA10353	f__LS-SOB	g__	
CO36405bin_22	d__Bacteria	p__Proteobacteria	c__Gammaproteobacteria	o__UBA6522	f__UBA6522	g__	
CO36388bin_2	d__Bacteria	p__Proteobacteria	c__Gammaproteobacteria	o__UBA11654	f__UBA11654	g__	
CO36386bin_20	d__Bacteria	p__Proteobacteria	c__Gammaproteobacteria	o__	f__	g__	
CO36386bin_8	d__Bacteria	p__Proteobacteria	c__Gammaproteobacteria	o__Coxiellales	f__Coxiellaceae	g__	
CO36388bin_10	d__Bacteria	p__Proteobacteria	c__Gammaproteobacteria	o__Pseudomonadales	f__HTCC2089	g__bin55	
CO36404bin_7	d__Bacteria	p__Proteobacteria	c__Gammaproteobacteria	o__Pseudomonadales	f__HTCC2089	g__bin55	

b) Macroalgae MAGs$_{95\%ANI}$

SS36439bin_8	d__Bacteria	p__Firmicutes	c__Bacilli	o__Bacillales	f__Bacillaceae	g__Bacillus	s__Bacillus licheniformis
SS36439bin_9	d__Bacteria	p__Firmicutes	c__Bacilli	o__Bacillales	f__Bacillaceae_A	g__Bacillus_AC	s__Bacillus_AC circulans_A
SS36440bin_1	d__Bacteria	p__Firmicutes	c__Bacilli	o__Bacillales	f__Bacillaceae_H	g__Bacillus_C	s__Bacillus_C aryabhattai_A
SS36439bin_7	d__Bacteria	p__Firmicutes	c__Bacilli	o__Bacillales	f__Bacillaceae	g__Bacillus	s__
SS36441bin_5	d__Bacteria	p__Firmicutes	c__Bacilli	o__Bacillales	f__Bacillaceae	g__Bacillus_AY	s__Bacillus_AY weihaiensis
SS36439bin_4	d__Bacteria	p__Firmicutes	c__Bacilli	o__Bacillales	f__Bacillaceae_H	g__Bacillus_C	s__Bacillus_C megaterium
SS36427bin_1	d__Bacteria	p__Chloroflexota	c__Anaerolineae	o__Promineofilales	f__	g__	s__
SS36439bin_2	d__Bacteria	p__Actinobacteriota	c__Actinobacteria	o__Actinomycetales	f__Demequinaceae	g__	s__
SS36427bin_3	d__Bacteria	p__Firmicutes	c__Bacilli	o__Bacillales	f__Bacillaceae_A	g__Bacillus_AC	s__
SS36439bin_11	d__Bacteria	p__Firmicutes	c__Bacilli	o__Bacillales	f__Bacillaceae_A	g__Bacillus_AK	s__
SS36440bin_2	d__Bacteria	p__Cyanobacteriota	c__Cyanobacteriia	o__Cyanobacteriales	f__Coleofasciculaceae	g__Moorea	s__
SS36425bin_4	d__Bacteria	p__Bacteroidota	c__Bacteroidia	o__Flavobacteriales	f__Flavobacteriaceae	g__Aquimarina	s__
SS36427bin_21	d__Bacteria	p__Bacteroidota	c__Bacteroidia	o__Flavobacteriales	f__Flavobacteriaceae	g__Aquimarina	s__
SS36425bin_2	d__Bacteria	p__Bacteroidota	c__Bacteroidia	o__Cytophagales	f__Amoebophilaceae	g__	s__
SS36425bin_5	d__Bacteria	p__Bacteroidota	c__Bacteroidia	o__Cytophagales	f__	g__	s__
SS36427bin_12	d__Bacteria	p__Verrucomicrobiota	c__Verrucomicrobiae	o__Verrucomicrobiales	f__Akkermansiaceae	g__	s__
SS36427bin_6	d__Bacteria	p__Spirochaetota	c__Spirochaetia	o__Spirochaetales	f__Alkalispirochaetaceae	g__	s__
SS36427bin_2	d__Bacteria	p__Proteobacteria	c__Alphaproteobacteria	o__Rhizobiales	f__	g__	s__
SS36439bin_6	d__Bacteria	p__Proteobacteria	c__Alphaproteobacteria	o__Sphingomonadales	f__Sphingomonadaceae	g__Altererythrobacter	s__
SS36439bin_13	d__Bacteria	p__Proteobacteria	c__Gammaproteobacteria	o__UBA10353	f__	g__	s__

c) Seawater MAGs$_{95\%ANI}$

PL34863bin_7	d__Archaea	p__Thermoplasmatota	c__MGII	o__MGII	f__MGIIB	g__UBA9562	s__GCA_002696615.1
PL34863bin_27	d__Archaea	p__Thermoplasmatota	c__MGII	o__MGII	f__MGIIB	g__UBA252	s__GCA_001628435.1
PL36323bin_28	d__Archaea	p__Asgardarchaeota	c__Heimdallarchaeia	o__LC-2	f__LC-2	g__GCA-2728275	s__GCA_002728275.1
PL34863bin_5	d__Archaea	p__Thermoplasmatota	c__MGII	o__MGII	f__MGIIB	g__UBA557	s__
PL34864bin_10	d__Archaea	p__Thermoplasmatota	c__MGII	o__MGII	f__MGIIB	g__UBA11751	s__
PL36325bin_14	d__Archaea	p__Thermoplasmatota	c__MGII	o__MGII	f__MGIIA	g__UBA562	s__
PL36329bin_3	d__Archaea	p__Thermoplasmatota	c__MGII	o__MGII	f__MGIIA	g__UBA253	s__
PL36325bin_29	d__Archaea	p__Thermoplasmatota	c__MGII	o__MGII	f__MGIIA	g__UBA120	s__
PL36311bin_9	d__Archaea	p__Thermoplasmatota	c__MGII	o__MGII	f__MGIIA	g__UBA120	s__
PL34865bin_12	d__Bacteria	p__Actinobacteriota	c__Acidimicrobiia	o__Microtrichales	f__UBA11606	g__MedAcidi-G2A	s__MedAcidi-G2A sp3
PL36330bin_20	d__Bacteria	p__Proteobacteria	c__Gammaproteobacteria	o__Pseudomonadales	f__Halieaceae	g__Luminiphilus	s__GCA_002703585.1
PL36330bin_32	d__Bacteria	p__Proteobacteria	c__Gammaproteobacteria	o__Pseudomonadales	f__Litoricolaceae	g__Litoricola	s__GCF_000227525.1
PL36311bin_20	d__Bacteria	p__Proteobacteria	c__Alphaproteobacteria	o__Rhodobacterales	f__Rhodobacteraceae	g__TMED111	s__TMED111 sp1
PL36325bin_24	d__Bacteria	p__Proteobacteria	c__Alphaproteobacteria	o__Rhodobacterales	f__Rhodobacteraceae	g__TMED111	s__
PL36325bin_8	d__Bacteria	p__Actinobacteriota	c__Acidimicrobiia	o__Microtrichales	f__UBA11606	g__UBA11606	s__GCA_002690205.1
PL36324bin_7	d__Bacteria	p__Proteobacteria	c__Alphaproteobacteria	o__Rhodobacterales	f__Rhodobacteraceae	g__HIMB11	s__GCA_001510135.1
PL36313bin_3	d__Bacteria	p__SAR324	c__SAR324	o__SAR324	f__NAC60-12	g__UBA1014	s__UBA1014 sp2
PL36323bin_1	d__Bacteria	p__Proteobacteria	c__Alphaproteobacteria	o__Puniceispirillales	f__Puniceispirillaceae	g__UBA8309	s__UBA8309 sp3
PL34863bin_47	d__Bacteria	p__Proteobacteria	c__Alphaproteobacteria	o__Nisaeales	f__Nisaeaceae	g__GCA-002701455	s__GCA_002690995.1
PL36312bin_6	d__Bacteria	p__Actinobacteriota	c__Acidimicrobiia	o__Microtrichales	f__Ilumatobacteraceae	g__Casp-actino5	s__
PL34863bin_39	d__Bacteria	p__Cyanobacteriota	c__Cyanobacteriia	o__Synechococcales_A	f__Cyanobiaceae	g__Synechococcus_C	s__
PL36324bin_3	d__Bacteria	p__Bacteroidota	c__Bacteroidia	o__Flavobacteriales	f__Cryomorphaceae	g__UBA10364	s__
PL36326bin_6	d__Bacteria	p__Bacteroidota	c__Bacteroidia	o__Flavobacteriales	f__Cryomorphaceae	g__UBA10364	s__
PL36329bin_53	d__Bacteria	p__Bacteroidota	c__Bacteroidia	o__Flavobacteriales	f__Flavobacteriaceae	g__MED-G14	s__
PL36327bin_25	d__Bacteria	p__Bacteroidota	c__Bacteroidia	o__Flavobacteriales	f__Flavobacteriaceae	g__MS024-2A	s__
PL36330bin_9	d__Bacteria	p__Bacteroidota	c__Bacteroidia	o__Flavobacteriales	f__Flavobacteriaceae	g__MS024-2A	s__
PL36330bin_11	d__Bacteria	p__Bacteroidota	c__Bacteroidia	o__Flavobacteriales	f__Flavobacteriaceae	g__MS024-2A	s__
PL36329bin_6	d__Bacteria	p__Bacteroidota	c__Bacteroidia	o__Flavobacteriales	f__Flavobacteriaceae	g__MS024-2A	s__
PL36324bin_14	d__Bacteria	p__Bacteroidota	c__Bacteroidia	o__Flavobacteriales	f__Flavobacteriaceae	g__MS024-2A	s__
PL36329bin_16	d__Bacteria	p__Bacteroidota	c__Bacteroidia	o__Flavobacteriales	f__Flavobacteriaceae	g__MS024-2A	s__
PL36329bin_2	d__Bacteria	p__Bacteroidota	c__Bacteroidia	o__Flavobacteriales	f__Flavobacteriaceae	g__MS024-2A	s__
PL36323bin_8	d__Bacteria	p__Bacteroidota	c__Bacteroidia	o__Flavobacteriales	f__Flavobacteriaceae	g__MS024-2A	s__
PL36326bin_18	d__Bacteria	p__Bacteroidota	c__Bacteroidia	o__Flavobacteriales	f__Flavobacteriaceae	g__BACL21	s__
PL36327bin_9	d__Bacteria	p__Bacteroidota	c__Bacteroidia	o__Flavobacteriales	f__Flavobacteriaceae	g__UBA3478	s__
PL36313bin_11	d__Bacteria	p__Bacteroidota	c__Bacteroidia	o__Flavobacteriales	f__UA16	g__UBA8752	s__
PL36328bin_37	d__Bacteria	p__Bacteroidota	c__Bacteroidia	o__Flavobacteriales	f__UA16	g__UBA11663	s__
PL36329bin_36	d__Bacteria	p__Bacteroidota	c__Bacteroidia	o__Flavobacteriales	f__UA16	g__UBA11663	s__
PL36312bin_4	d__Bacteria	p__Bacteroidota	c__Bacteroidia	o__Flavobacteriales	f__Crocinitomicaceae	g__UBA952	s__
PL36313bin_23	d__Bacteria	p__Bacteroidota	c__Bacteroidia	o__Flavobacteriales	f__UBA10066	g__	s__
PL34864bin_44	d__Bacteria	p__Marinisomatota	c__Marinisomatia	o__Marinisomatales	f__Marinisomataceae	g__Marinisoma	s__
PL36330bin_18	d__Bacteria	p__Planctomycetota	c__UBA1135	o__UBA1135	f__GCA-002686595	g__GCA-2686945	s__
PL36329bin_1	d__Bacteria	p__Planctomycetota	c__UBA8108	o__UBA1146	f__UBA1146	g__UBA12191	s__
PL36328bin_29	d__Bacteria	p__Planctomycetota	c__Planctomycetes	o__Pirellulales	f__Pirellulaceae	g__Rubripirellula	s__
PL36313bin_20	d__Bacteria	p__Verrucomicrobiota	c__Verrucomicrobiae	o__Opitutales	f__Puniceicoccaceae	g__GCA-2690565	s__
PL34863bin_23	d__Bacteria	p__Verrucomicrobiota	c__Verrucomicrobiae	o__Pedosphaerales	f__UBA1096	g__UBA1096	s__
PL34863bin_20	d__Bacteria	p__Verrucomicrobiota	c__Verrucomicrobiae	o__Pedosphaerales	f__UBA1100	g__UBA1100	s__
PL34863bin_31	d__Bacteria	p__Verrucomicrobiota	c__Verrucomicrobiae	o__Pedosphaerales	f__	g__	s__
PL34863bin_32	d__Bacteria	p__Verrucomicrobiota	c__Verrucomicrobiae	o__Verrucomicrobiales	f__DEV007	g__EC70	s__
PL36313bin_19	d__Bacteria	p__SAR324	c__SAR324	o__SAR324	f__NAC60-12	g__JCVI-SCAAA005	s__
PL36325bin_39	d__Bacteria	p__Proteobacteria	c__Alphaproteobacteria	o__Pelagibacterales	f__Pelagibacteraceae	g__Pelagibacter	s__
PL36310bin_3	d__Bacteria	p__Proteobacteria	c__Alphaproteobacteria	o__Puniceispirillales	f__UBA1172	g__	s__
PL36330bin_25	d__Bacteria	p__Proteobacteria	c__Alphaproteobacteria	o__Puniceispirillales	f__Puniceispirillaceae	g__UBA8309	s__
PL36329bin_7	d__Bacteria	p__Proteobacteria	c__Alphaproteobacteria	o__Puniceispirillales	f__Puniceispirillaceae	g__	s__
PL36310bin_7	d__Bacteria	p__Proteobacteria	c__Alphaproteobacteria	o__Puniceispirillales	f__Puniceispirillaceae	g__	s__
PL36329bin_29	d__Bacteria	p__Proteobacteria	c__Alphaproteobacteria	o__Puniceispirillales	f__Puniceispirillaceae	g__	s__
PL36308bin_32	d__Bacteria	p__Proteobacteria	c__Alphaproteobacteria	o__Puniceispirillales	f__Puniceispirillaceae	g__	s__
PL36326bin_7	d__Bacteria	p__Proteobacteria	c__Alphaproteobacteria	o__Puniceispirillales	f__Puniceispirillaceae	g__	s__
PL36308bin_10	d__Bacteria	p__Proteobacteria	c__Alphaproteobacteria	o__Puniceispirillales	f__Puniceispirillaceae	g__	s__
PL36324bin_21	d__Bacteria	p__Proteobacteria	c__Alphaproteobacteria	o__Rhodobacterales	f__Rhodobacteraceae	g__HIMB11	s__
PL34864bin_26	d__Bacteria	p__Proteobacteria	c__Gammaproteobacteria	o__SAR86	f__D2472	g__	s__
PL36313bin_60	d__Bacteria	p__Proteobacteria	c__Gammaproteobacteria	o__SAR86	f__SAR86	g__GCA-2707915	s__
PL34864bin_11	d__Bacteria	p__Proteobacteria	c__Gammaproteobacteria	o__UBA10353	f__LS-SOB	g__UBA5682	s__
PL36328bin_12	d__Bacteria	p__Proteobacteria	c__Gammaproteobacteria	o__Xanthomonadales	f__	g__	s__
PL36313bin_15	d__Bacteria	p__Proteobacteria	c__Gammaproteobacteria	o__Pseudomonadales	f__Pseudohongiellaceae	g__UBA9145	s__
PL36323bin_13	d__Bacteria	p__Proteobacteria	c__Gammaproteobacteria	o__Pseudomonadales	f__Halieaceae	g__Luminiphilus	s__
PL36313bin_41	d__Bacteria	p__Proteobacteria	c__Gammaproteobacteria	o__Pseudomonadales	f__Halieaceae	g__Luminiphilus	s__
PL34863bin_51	d__Bacteria	p__Proteobacteria	c__Gammaproteobacteria	o__Pseudomonadales	f__Halieaceae	g__Luminiphilus	s__

Supplementary Table S4. Statistical output of differential abundance analysis (DESeq) highlighting significantly enriched microbial phyla (class for Proteobacteria) in a) sponge, b) macroalgae, and c) seawater microbiomes between winter and summer.

a) Sponge microbiome

phylum.class	baseMean	log2FoldChange	lfcSE	stat	pvalue	padj
p__Chloroflexota	16850339	-3.993485	0.844069	-4.73123	2.23E-06	2.45E-05

b) macroalgae microbiome

phylum.class	baseMean	log2FoldChange	lfcSE	stat	pvalue	padj
c__Alphaproteobacteria	276990.52	-2.311277	0.6133642	-3.768197	1.64E-04	2.47E-04
c__Gammaproteobacteria	43427.11	19.48362	1.0783512	18.067973	5.70E-73	2.56E-72
p__Actinobacteriota	47004.16	1.526713	0.6728611	2.268986	2.33E-02	2.33E-02
p__Bacteroidota	488327.3	-9.193737	2.2798199	-4.032659	5.51E-05	9.93E-05
p__Chloroflexota	81121.98	-7.093359	2.6293516	-2.69776	6.98E-03	8.98E-03
p__Cyanobacteriota	466645.65	22.769968	1.2215184	18.640709	1.50E-77	1.35E-76
p__Firmicutes	12566031.97	6.072401	2.4164859	2.512906	1.20E-02	1.35E-02
p__Spirochaetota	28686.83	-30	3.9067681	-7.678982	1.60E-14	3.61E-14
p__Verrucomicrobiota	64297.23	-18.980284	1.3903511	-13.651432	1.98E-42	5.94E-42

c) seawater

phylum.class	baseMean	log2FoldChange	lfcSE	stat	pvalue	padj
c__Alphaproteobacteria	3235902	-0.3797838	0.1126066	-3.372661	7.44E-04	1.17E-03
p__Bacteroidota	4718048.6	1.0013896	0.1693244	5.914031	3.34E-09	1.16E-08
p__Cyanobacteriota	140817.2	-1.1383357	0.2267763	-5.019642	5.18E-07	1.14E-06
p__Marinisomatota	303744.4	1.1451393	0.2831123	4.044823	5.24E-05	9.60E-05
p__Planctomycetota	347855.5	3.1608062	0.1561974	20.235969	4.72E-91	5.19E-90
p__SAR324	180286.2	0.7702925	0.1085726	7.094722	1.30E-12	7.13E-12
p__Thermoplasmatota	674037	-0.4235043	0.151861	-2.788762	5.29E-03	7.28E-03
p__Verrucomicrobiota	325162.6	-0.6196964	0.1054822	-5.874888	4.23E-09	1.16E-08

Table S5. Planktonic Bacteriodota MAGs$_{95\%ANI}$ and the presence of GH families (CAZy database).

Bin_ID	GH16	GH37	GH33	GH30_1	GH13	GH17	GH29	GH53	GH5	GH2	GH95	GH18	GH1	GH30	GH65	GH97	GH113	GH63	GH73	GH13_36	GH43_18	GH5_13
PL36312bin_4	0	0	0	0	0	0	0	0	0	0	0	0	1	1	1	0	0	0	0	0	0	0
PL36313bin_11	0	0	0	0	0	0	0	1	0	2	0	0	0	0	0	0	0	0	0	0	0	0
PL36313bin_23	0	0	0	0	0	0	0	0	0	1	0	0	1	0	0	0	0	0	1	0	0	0
PL36323bin_8	2	0	1	0	0	0	2	0	0	0	0	1	0	0	0	0	0	0	0	0	0	0
PL36324bin_14	1	0	1	0	0	0	1	0	0	0	0	0	0	0	0	0	0	0	0	0	0	1
PL36324bin_3	0	0	1	1	1	2	0	1	1	0	0	0	0	0	0	0	0	0	0	0	0	0
PL36326bin_18	0	0	0	1	1	2	0	0	0	0	0	0	0	0	0	0	0	0	0	0	0	0
PL36326bin_6	0	1	0	1	0	0	0	1	1	0	0	0	0	0	0	1	0	0	0	0	0	0
PL36327bin_25	2	1	0	1	1	0	0	0	0	0	1	0	0	0	1	0	0	0	0	0	0	0
PL36327bin_9	1	0	1	0	0	0	1	0	0	0	0	0	0	1	0	0	1	0	0	0	1	0
PL36328bin_37	0	0	0	0	0	0	0	0	0	0	0	0	0	0	0	0	0	0	0	0	0	0
PL36329bin_16	2	1	0	1	1	0	0	1	0	0	0	0	0	0	0	1	0	1	0	0	0	0
PL36329bin_2	2	1	0	0	0	0	0	0	0	0	0	0	0	0	0	0	0	0	0	0	0	0
PL36329bin_36	0	0	1	0	0	0	0	0	0	0	0	0	0	0	0	0	0	0	0	0	0	0
PL36329bin_53	1	0	0	0	0	0	0	0	0	0	0	1	0	0	0	0	0	0	0	0	0	0
PL36329bin_6	1	1	0	0	0	0	0	0	0	0	1	0	0	0	0	0	0	0	0	0	0	0
PL36330bin_11	1	0	0	0	0	0	0	0	1	0	0	0	0	0	0	0	0	0	0	1	0	0
PL36330bin_9	2	1	0	0	0	0	0	0	1	0	1	0	0	0	0	0	0	0	0	0	0	0

Table S6. Planktonic Bacteriodota MAGs$_{95\%ANI}$ and the presence of SusD-like genes (Pfam database).

Bin_ID	PF07980.10	PF12741.6	PF14322.5	PF12771.6
**PL36312bin_4**	2	0	2	0
**PL36313bin_11**	2	1	3	2
**PL36313bin_23**	2	1	2	1
**PL36323bin_8**	1	2	3	2
**PL36324bin_14**	4	1	5	2
**PL36324bin_3**	2	1	3	1
**PL36326bin_18**	2	1	2	1
**PL36326bin_6**	4	3	6	3
**PL36327bin_25**	5	0	6	3
**PL36327bin_9**	3	1	3	1
**PL36328bin_37**	2	0	1	0
**PL36329bin_16**	8	1	7	3
**PL36329bin_2**	5	1	4	2
**PL36329bin_36**	2	0	2	0
**PL36329bin_53**	1	1	2	1
**PL36329bin_6**	4	2	5	2
**PL36330bin_11**	2	2	5	3
**PL36330bin_9**	4	2	4	2

Table S7. Presence of MAGs$_{95\%ANI}$ in seawater, macroalgae and sponge samples. Adapter-trimmed reads from samples collected in August 2016 and February 2017 were mapped (75% minimum alignment and 95% minimum identity) against the de-replicated MAGs$_{95\%ANI}$ with coverM v0.2.0 (https://github.com/wwood/CoverM). Nine sponge MAGs$_{95\%ANI}$ were present in seawater and macroalgae samples, 14 seawater MAGs$_{95\%ANI}$ were present in sponge and macroalgae samples and no macroalgae MAGs$_{95\%ANI}$ were found in seawater and sponge samples.

							seawater samples						macroalgae samples					
Sponge MAGs	Domain	Phylum	Class	Order	Family	Genus	Aug-16	Aug-16	Aug-16	Feb-17	Feb-17	Feb-17	Aug-16	Aug-16	Aug-16	Feb-17	Feb-17	Feb-17
CO36386bin_10	d__Bacteria	p__Planctomycetota	c__Planctomycetes	o__Pirellulales	f__Pirellulaceae	g__Mariniblastus	1	1	1	0	0	0	1	0	1	0	0	0
CO36386bin_29	d__Bacteria	p__Cyanobacteriota	c__Cyanobacteriia	o__Synechococcales_A	f__Cyanobiaceae	g__Synechococcus_C	1	1	1	1	1	1	0	0	1	0	0	0
CO36386bin_37	d__Bacteria	p__Cyanobacteriota	c__Cyanobacteriia	o__Synechococcales_A	f__Cyanobiaceae	g__Synechococcus_C	1	1	1	0	0	0	0	0	0	0	0	0
CO36386bin_8	d__Bacteria	p__Proteobacteria	c__Gammaproteobacteria	o__Coxiellales	f__Coxiellaceae	g__	1	1	1	0	0	0	0	0	0	0	0	0
CO36386bin_9	d__Bacteria	p__Proteobacteria	c__Alphaproteobacteria	o__bin65	f__bin65	g__bin65	0	0	0	0	0	0	0	0	0	0	0	0
CO36387bin_4	d__Bacteria	p__Planctomycetota	c__Planctomycetes	o__Pirellulales	f__Pirellulaceae	g__Rubripirellula	1	1	1	1	1	1	1	1	1	0	0	0
CO36388bin_9	d__Bacteria	p__Acidobacteriota	c__Thermoanaerobaculia	o__	f__	g__	0	0	0	0	0	0	1	0	1	0	0	0
CO36405bin_2	d__Bacteria	p__Planctomycetota	c__Planctomycetes	o__Pirellulales	f__UBA1268	g__UBA1268	1	1	1	1	1	1	0	0	1	0	0	0
CO36406bin_26	d__Bacteria	p__Cyanobacteriota	c__Cyanobacteriia	o__Synechococcales_A	f__Cyanobiaceae	g__Synechococcus_C	1	1	1	1	1	1	0	0	0	0	0	0

							sponge samples						macroalgae samples					
Seawater MAGs	Domain	Phylum	Class	Order	Family	Genus	Aug-16	Aug-16	Aug-16	Feb-17	Feb-17	Feb-17	Aug-16	Aug-16	Aug-16	Feb-17	Feb-17	Feb-17
PL34863bin_32	d__Bacteria	p__Verrucomicrobiota	c__Verrucomicrobiae	o__Verrucomicrobiales	f__DEV007	g__EC70	1	1	0	0	0	0	1	1	1	0	0	0
PL34863bin_39	d__Bacteria	p__Cyanobacteriota	c__Cyanobacteriia	o__Synechococcales_A	f__Cyanobiaceae	g__Synechococcus_C	1	1	1	1	1	1	0	0	1	0	0	0
PL34863bin_51	d__Bacteria	p__Proteobacteria	c__Gammaproteobacteria	o__Pseudomonadales	f__Halieaceae	g__Luminiphilus	1	0	0	0	0	0	0	0	0	0	0	0
PL36312bin_4	d__Bacteria	p__Bacteroidota	c__Bacteroidia	o__Flavobacteriales	f__Crocinitomicaceae	g__UBA952	0	0	0	0	0	0	1	1	1	0	0	0
PL36312bin_6	d__Bacteria	p__Actinobacteriota	c__Acidimicrobiia	o__Microtrichales	f__Ilumatobacteraceae	g__Casp-actino5	1	1	0	0	0	0	0	0	0	0	0	0
PL36313bin_11	d__Bacteria	p__Bacteroidota	c__Bacteroidia	o__Flavobacteriales	f__UA16	g__UBA8752	0	0	0	0	0	0	1	1	1	0	0	0
PL36313bin_15	d__Bacteria	p__Proteobacteria	c__Gammaproteobacteria	o__Pseudomonadales	f__Pseudohongiel	g__UBA9145	0	0	0	0	0	0	0	0	0	0	0	0
PL36313bin_19	d__Bacteria	p__SAR324	c__SAR324	o__SAR324	f__NAC60-12	g__JCVI-SCAAA005	1	1	0	0	1	1	0	0	0	0	0	0
PL36323bin_13	d__Bacteria	p__Proteobacteria	c__Gammaproteobacteria	o__Pseudomonadales	f__Halieaceae	g__Luminiphilus	0	0	0	0	0	0	1	1	1	1	0	0
PL36324bin_7	d__Bacteria	p__Proteobacteria	c__Alphaproteobacteria	o__Rhodobacterales	f__Rhodobacteraceae	g__HIMB11	1	0	0	0	0	0	0	0	0	0	0	0
PL36327bin_9	d__Bacteria	p__Bacteroidota	c__Bacteroidia	o__Flavobacteriales	f__Flavobacteriaceae	g__UBA3478	0	0	0	0	0	0	1	1	1	1	0	1
PL36328bin_12	d__Bacteria	p__Proteobacteria	c__Gammaproteobacteria	o__Xanthomonadales	f__	g__	0	0	0	1	1	1	1	1	1	1	1	1
PL36328bin_29	d__Bacteria	p__Planctomycetota	c__Planctomycetes	o__Pirellulales	f__Pirellulaceae	g__Rubripirellula	1	1	0	1	1	1	0	0	1	1	0	0
PL36329bin_53	d__Bacteria	p__Bacteroidota	c__Bacteroidia	o__Flavobacteriales	f__Flavobacteriaceae	g__MED-G14	0	0	0	0	0	0	1	1	1	0	0	0
